# How conditions within immigration removal centres in the United Kingdom affect the mental wellbeing of detainees: a scoping review

**DOI:** 10.1093/pubmed/fdag044

**Published:** 2026-06-26

**Authors:** Niamh Gallagher, Tristan Price, Matthew Griggs

**Affiliations:** Department of Clinical and Biomedical Sciences, University of Exeter Medical School (Faculty of Health and Life Sciences), Knowledge Spa, Royal Cornwall Hospital, Penventinnie Lane, Truro, Cornwall TR1 3HD, UK; Peninsula Medical School (Faculty of Health), University of Plymouth, The Faculty of Medicine and Dentistry, The John Bull Building, Plymouth Science Park, Research Way, Plymouth, Devon PL6 8BU, UK; Peninsula Medical School (Faculty of Health), University of Plymouth, The Faculty of Medicine and Dentistry, The John Bull Building, Plymouth Science Park, Research Way, Plymouth, Devon PL6 8BU, UK

**Keywords:** mental health, migration, public health

## Abstract

**Background:**

Immigration Removal Centres (IRCs) are used to detain people for immigration control by the UK government. This scoping review aims to examine the experiences of detainees within UK IRCs, specifically how conditions within them, including the regime, affect their mental wellbeing.

**Methods:**

The Joanna Briggs Institute guideline for scoping reviews was followed. Six bibliographic databases and additional grey literature sources were searched for quantitative and qualitative evidence. Descriptive analyses and quality assessments were conducted.

**Results:**

Fifteen research studies and nine pieces of grey literature were included, comprising a total of 1353 participants and 11 IRCs. The majority of data was qualitative in methodology and published after 2015. Main findings from articles were charted according to Maslow’s (1943) Hierarchy of Needs, revealing persistent failings across all dimensions (physiological, safety, belongingness, esteem, and self-actualization). The regime within the IRCs as well as the psychosocial environment led to emotional distress and feelings of disempowerment, dehumanization, and criminality.

**Conclusions:**

This review highlights the negative impact of IRCs within the UK on the mental wellbeing of detainees and the need for urgent policy reform. Changes addressing temporal uncertainty of detention and use of community-based settings are proposed for UK governmental review.

## Introduction

Immigration detention is the administrative practice of detaining foreign nationals for the purpose of resolving their immigration status.^1^ The UK Home Office states that detention may be required due to uncertainties around temporary admission or release, pending imminent removal, whilst awaiting care arrangements or where release is not considered conducive to the public good.^2^ Detention occurs in Immigration Removal Centres (IRCs), short-term holding facilities (STHFs), pre-departure accommodation, or prisons. In the year ending March 2025, 20 919 people entered UK immigration detention.^3^ At the time of this review in August 2023, UK immigration detention consisted of seven IRCs and four residential STHFs, managed by private providers and the prison service (Supplementary File SA).

His Majesty’s Inspectorate of Prisons (HMIP) is an independent body responsible for human rights-based monitoring of standards across all detention facilities—including criminal, immigration, police, court, and military settings—through inspections conducted in accordance with the Detention Centre Rules (2001).^4^ By evaluating conditions, legal compliance, and procedural safeguards, HMIP provides authoritative oversight, highlights gaps between policy and practice, and contributes to the protection of detainees’ rights and the improvement of detention practices.

The treatment and subsequent wellbeing of detainees within IRCs has long been the subject of sustained criticism from policymakers, civil society organizations and the public.^2^^,^^5–8^ In 2016, Stephen Shaw’s independent review made 64 specific recommendations after finding detention intrinsically harmful, especially for vulnerable individuals, and that safeguards such as Rule 35 were ineffective.^9^ Shaw highlighted that vulnerability is dynamic and exacerbated by uncertainty and prolonged detention, recommending reductions in detention scale and greater use of community-based alternatives.^9^

Five IRCs closed between 2015 and 2021, and two new IRCs (Brook House and Derwentside) opened in 2009 and 2021, respectively.^1^^,^^10^ Legally, there is no time limit on detention, and despite the Immigration Act (2016) introducing automatic bail hearings, 29% of detainees in the year ending June 2023 spent over 28 days in detention.^3^ This Act has been superseded, with improvements including those prompted by the Windrush Scandal (such as staff training in immigration history and equality, a more ethical and clear purpose, mission and values, and risk management strategies) negated by the Illegal Migration Act (2023).^11–13^ The new bill shifts towards a more punitive and restrictive approach to immigration enforcement and reassigns powers relating to detention, including time restrictions, conditions in detention and legal rights of detainees, from the courts to the Home Office.^14^

Concerns regarding welfare and accountability were further intensified by the Brook House Inquiry (2023), a statutory public inquiry into mistreatment at Brook House IRC.^15^ The Inquiry identified 19 incidents capable of amounting to inhuman or degrading treatment under Article 3 of the European Convention on Human Rights, including excessive use of force, dangerous restraint techniques, humiliating treatment during self-harm episodes, and racist or derogatory language by staff. Crucially, it concluded that these harms arose from systemic failures in oversight, contract management, safeguarding processes and institutional culture, rather than isolated acts of misconduct.^15^

International evidence, largely concerning both on- and off-shore Australian immigration detention centres, indicates high levels of mental health issues in individuals in immigration detention—most commonly depression, anxiety, and post-traumatic stress disorder (PTSD).^16–19^ Although the causes of these mental health conditions are likely multifactorial, research has identified a link between prolonged detention and adverse mental health outcomes, reinforced through longitudinal studies.^18^^,^^20–22^ In the UK, the Centre for Mental Health asserts that every detainee is exposed to mental health or well-being challenges, experiencing psychological and emotional distress, even if they do not meet a clinical threshold.^23^

The Home Office detention instructions state that detention must be used sparingly, and only for the shortest period necessary.^24^ Similarly, the United Nations advises that detention should only be employed solely as a measure of last resort for asylum-seekers.^25^ However, in 2012 HMIP identified significant legality concerns in relation to both initial detention decisions and the subsequent review process.^26^ These included, but were not limited to, poorly informed decision-making; a lack of coherence in reviews and inter-professional communication; barriers to accessing good-quality legal advice; the normalized automatic detention of Foreign National Offenders (FNOs) following release from prison; and casework inefficiencies resulting in prolonged detention. Collectively, these findings suggest a systematic disconnect between the stated legal safeguards governing detention and their implementation in practice.

As the UK continues to detain immigrants and tighten regulations in this area, this review was conducted with the aim of identifying which conditions within IRCs in the UK are impacting the mental wellbeing of detainees and how. It utilizes Maslow’s (1943) Hierarchy of Needs to structure thematic analysis due to its broad categories pertaining to health and wellbeing.^27^

## Methods

The guideline for scoping reviews from the Joanna Briggs Institute (JBI) Manual for Evidence Synthesis was consulted, as well as guidelines for conducting a systematic review of qualitative evidence.^28^^,^^29^

### Inclusion criteria

Populations of interest were adults who had been or were currently detained for immigration purposes for any length of time (Supplementary File SB). This included but was not limited to foreign national offenders, asylum seekers, and those who overstayed or breached the terms of their visa. The phenomena of interest were the experiences of conditions within detention affecting detainees’ mental wellbeing. Context was limited to IRCs in the UK, however articles which contained STHF and pre-departure accommodation were included if unable to be delineated from the IRC. Primary quantitative and qualitative research from published and grey literature, published in English between 1 January 2001 (introduction of the Detention Centre Rules) and 30 August 2023, was examined.

### Information sources and search strategy

Six bibliographic databases were searched via their corresponding platforms of EBSCO (CINAHL Plus with Full Text, MEDLINE, SocINDEX with Full Text and Global Health), OVID (Embase), and ProQuest (PsycINFO). Search terms were derived from the PICo framework (Table 1) and corresponding database thesaurus terms were included (Supplementary File SC). Grey literature was identified through searches of online databases and websites of UK governmental and non-governmental organizations relating to IRCs. Forward citation searches were conducted of eligible articles for additional evidence sources.

**Table 1:** PICo framework.

Participants	Adults (over 18 years old) who have been, or are currently, detained for immigration administrative purposes (including but not limited to foreign national offenders and asylum seekers)
Phenomena of Interest	Experiences of conditions of detention affecting their mental wellbeing
Context	IRCs located within the UK

### Study selection and data extraction

Citations from bibliographic database searches were imported into EndNote, and duplicates were removed. Articles were screened according to their titles and/or abstract and excluded if not meeting eligibility criteria, followed by screening of full texts (Supplementary File SD). Data were manually extracted, with key study findings charted according to Maslow’s Hierarchy of Needs^27^ (Supplementary File SE).

### Data synthesis and analysis

Qualitative data from key findings were inductively coded and themed according to Maslow’s Hierarchy of Needs^27^ (Table 2). This theory of human motivation was utilized due to its practical organization and interdisciplinary relevance, making it particularly valuable in the complex environment of IRCs where political management and health are inextricably intertwined. It is also hoped that its common prevalence provides policymakers with a recognizable format, increasing accessibility for identification of areas for improvement. It has been applied in multidisciplinary settings (sociology, education, management, and public health) as well as diverse cultural and global contexts, whilst acknowledging the order and density of each need varies accordingly.^30–33^ Therefore, the concept of a hierarchy was not utilized given its probable inconsistencies between populations.

**Table 2:** Summary table of codes generated from study results according to Maslow’s Hierarchy of Needs (Maslow, 1943).

*Theme*	*Subtheme*	*Code*	*Supporting references*	*Conflicting references*
**Basic needs**	**Basic**	Issues with food including quality, dietary choice, hunger strikes and strict mealtimes or restricted locations for eating	^33–40^ ^,^ ^46–49^	^31^ ^,^ ^32^^,^^38^^,^^45^
		Sleep quality impacted by noise from staff or other detainees, staff monitoring, and night-time transfers	^17^ ^,^ ^38–42^^,^^47–49^	^38^
		Poor living conditions including cleanliness and clothes	^17^ ^,^ ^37^^,^^43^^,^^48^	^31^ ^,^ ^38^^,^^45^^,^^46^
		Loss of possessions causing feelings of indignity	^41^ ^,^ ^47–49^	
		Issues with temperature control of detainee rooms	^38^ ^,^ ^39^^,^^48^	^46^
	**Safety**	Fear of physical or emotional abuse from both staff and other detainees	^17^ ^,^ ^33^^,^^39^^,^^41^^,^^44^^,^^48^^,^^49^	^48^
		Inadequate healthcare provision both physical and mental	^17^ ^,^ ^31–38^^,^^43^^,^^44^^,^^46^^,^^48^^,^^50–52^	^17^ ^,^ ^38^^,^^46^^,^^48^
		Inadequate access to medications due to supply issues and temporary discontinuation of medication on arrival	^33^ ^,^ ^42^^,^^44^^,^^50^^,^^51^	
		Fear of persecution due to gender, sexual orientation, nationality, or race	^17^ ^,^ ^31^^,^^37^^,^^39^^,^^44^^,^^48^^,^^51^	
		Distress of witnessing other detainee’s distress and re-traumatization	^33^ ^,^ ^35^^,^^43^^,^^44^^,^^47^^,^^49^^,^^50^	
		Difficulties accessing legal support/information	^36^ ^,^ ^38^^,^^39^^,^^41^^,^^43^^,^^44^^,^^46–49^^,^^52^	^38^ ^,^ ^48^
		Drug and alcohol use	^17^ ^,^ ^37^	
		Use of separation unit increased isolation and paranoia	^17^ ^,^ ^46^^,^^48^	
		Lack of pre-detention, inter-detention, and post-detention communication, and safeguards including transition of care	^17^ ^,^ ^36^^,^^38^^,^^41^^,^^44^^,^^46^^,^^49^	
**Psychological needs**	**Belongingness**	Challenges to national and cultural identity	^39^ ^,^ ^40^	
		Social isolation due to barriers for interaction between detainees including language, distress, and transiency	^17^ ^,^ ^31^^,^^37^^,^^39^^,^^40^^,^^48^^,^^49^	^41^
		Separation from family causing distress, anxiety, and childcare challenges	^47–49^	
		Inadequate provision or use of diversity and inclusion adjustments	^33^ ^,^ ^35–38^^,^^43^^,^^46^^,^^48^	
		Lack of communication and emotional support from staff	^17^ ^,^ ^33^^,^^35^^,^^38^^,^^39^^,^^42^^,^^46^^,^^47^^,^^51^^,^^54^	
		Challenged communication and support from the ‘outside world’ including financial/geographical barriers, visitor restrictions, monitored conversations, and phone and social media restrictions	^37–40^ ^,^ ^42^^,^^44^^,^^46–49^	^31^
		Coping strategies through faith, philosophy, activities, and social support	^17^ ^,^ ^35^^,^^39^^,^^42^^,^^45^^,^^48^^,^^49^	
		Bilateral mistrust between staff and detainees	^17^ ^,^ ^34^^,^^37^^,^^39^^,^^40^^,^^48^^,^^54^	^48^
	**Esteem**	Loss of agency such as over one’s environment and daily routine	^17^ ^,^ ^39^^,^^45^	
		Feeling criminalized	^17^ ^,^ ^38^^,^^39^^,^^42^^,^^47–49^^,^^51^^,^^52^	
		Challenges to self-esteem through perceived illegitimacy, discrimination and stigmatization	^33^ ^,^ ^35^^,^^39^^,^^40^^,^^42–44^^,^^48–50^^,^^52^^,^^54^	
		Hierarchy of power open to abuse and initiating feelings of powerlessness	^17^ ^,^ ^33^^,^^35^^,^^38^^,^^39^^,^^42^^,^^44^^,^^49^^,^^51^^,^^54^	
		Feelings of socio-political inequality and injustice	^45^ ^,^ ^47–49^^,^^54^	
		Lack of respect with invasions of privacy	^17^ ^,^ ^33^^,^^34^^,^^37–39^^,^^42–44^^,^^46^	^38^ ^,^ ^46^
		Dehumanization	^39^ ^,^ ^47^^,^^48^^,^^52^	
		Challenges to self-identity	^33^ ^,^ ^35^^,^^37^^,^^39^^,^^42^^,^^48^^,^^52^	
**Self-actualization**		Temporal uncertainty restricting one’s ability to plan for the future	^17^ ^,^ ^39^^,^^40^^,^^43^^,^^45^^,^^47–49^^,^^52^	
		Limited or poor-quality educational opportunities	^31^ ^,^ ^37^^,^^38^^,^^46^	
		Perceived loss of purpose or meaning to life	^17^ ^,^ ^35^^,^^45^	

Quantitative data were presented descriptively due to a scarcity of data.

### Quality analysis

Corresponding JBI critical appraisal tools were used for qualitative, prevalence, cross-sectional, and evidence synthesis studies (or the equivalent components of mixed-methods studies).^34–37^ Grey literature was classified according to the Oxford Centre for Evidence-Based Medicine (OCEBM) Levels of Evidence guidance.^38^ Studies were not excluded based on quality (Supplementary File SF).

## Results

### Characteristics of included articles

Fifteen studies and nine pieces of grey literature were included in the review. Only the two most recent HMIP inspections of IRCs (Tinsley House and Derwentside) were included. Of the total 24 articles, 13 were qualitative studies, 2 quantitative, 6 mixed methods, 2 evidence synthesis, and 1 leaflet. Study characteristics are displayed in Table 3. Where data sets were replicated between articles, only one set of population characteristics was included.^39–43^ Most articles were published between 2015 and 2023 (*n* = 20). Eleven articles specified where their study population was, or had been, residing, spanning a total of 11 different IRCs. Three of these have since closed (Morton Hall, Dover and The Verne) and Campsfield House closed but later re-opened. Where specified, total numbers of detainees equalled 1353. The majority of these were current detainees (*n* = 616/740) and/or asylum seekers (*n* = 64/82). Where gender and sexual orientation were identified, 74% were male (*n* = 405/545), 26% were female (*n* = 140/545), and 22 detainees identified as Lesbian, Gay, Bisexual, Transgender, Queer or any other sexual orientation or gender identity (LGBTQ+). Nine articles specified detainee’s country/continent of origin with a total of 25 different places. Detainees ranged between 19 and 82 years old, and time in detention ranged between 1 day and 2 years 8 months. Thirteen articles included staff members or other stakeholders, such as managers, practitioners or members of non-governmental organizations, as participants.

**Table 3:** Study characteristics.

Study characteristics			Numbers	References
**Year of publication (No. of studies)**	**2001–2009**		0	
	**2010–2014**		4	^41^ ^,^ ^48–50^
	**2015–2019**		11	^17^ ^,^ ^31^^,^ ^34^^,^ ^35^^,^ ^39^^,^ ^42–45^^,^ ^47^^,^ ^51^
	**2020–2023**		9	^32^ ^,^ ^33^^,^ ^36–38^^,^ ^40^^,^ ^46^^,^ ^52^^,^ ^54^
**Types of studies (No. of studies)**	**Qualitative**		13	^34^ ^,^ ^35^^,^ ^39–41^^,^ ^43–45^^,^ ^47^^,^ ^49^^,^ ^51^^,^ ^52^^,^ ^54^
	**Quantitative**		2	^31^ ^,^ ^32^
	**Mixed methods**		6	^17^ ^,^ ^37^^,^ ^38^^,^ ^46^^,^ ^48^^,^ ^50^
	**Evidence synthesis**		2	^33^ ^,^ ^36^
	**Grey**		1	^42^
**IRC studied (No. of studies)**	**Brook House**		4	^17^ ^,^ ^37^^,^ ^40^^,^ ^48^
	**Tinsley House**		5	^17^ ^,^ ^37^^,^ ^38^^,^ ^40^^,^ ^48^
	**Yarl's Wood**		6	^17^ ^,^ ^37^^,^ ^39^^,^ ^45^^,^ ^48^^,^ ^52^
	**Colnbrook**		4	^17^ ^,^ ^37^^,^ ^45^^,^ ^48^
	**Harmondsworth**		3	^17^ ^,^ ^37^^,^ ^39^
	**Dungavel**		2	^35^ ^,^ ^37^
	**Dover**		1	^45^
	**Morton Hall**		3	^17^ ^,^ ^37^^,^ ^48^
	**The Verne**		1	^17^
	**Campsfield House**		5	^17^ ^,^ ^41^^,^ ^45^^,^ ^48^^,^ ^49^
	**Derwentside**		1	^46^
	**N/S**		13	^31–34^ ^,^ ^36^^,^ ^39^^,^ ^42–44^^,^ ^47^^,^ ^50^^,^ ^51^^,^ ^54^
**Population characteristics**	**Numbers of detainees**	**Total**	1353 (No. N/S - 4 Studies)	^17^ ^,^ ^31^^,^ ^34–41^^,^ ^43–47^^,^ ^49–52^^,^ ^54^
		**Gender/Sexuality**	140 Women 405 Men 22 LGBTQ+ 786 N/S	
		**Detention status**	616 Current 124 Former	^17^ ^,^ ^31^^,^ ^34^^,^ ^35^^,^ ^38–41^^,^ ^44^^,^ ^46^^,^ ^47^^,^ ^49^^,^ ^50^^,^ ^52^
		**Asylum seekers/FNOs**	64 Asylum seekers 16 FNOs 2 Other	^35^ ^,^ ^39^^,^ ^44^^,^ ^47^^,^ ^50^
	**Ages (years old)**		19–82	^31^ ^,^ ^35^^,^ ^37^^,^ ^39^^,^ ^46^^,^ ^50^
	**Countries and/or continents of origin**	Afghanistan Africa Asia Benin Caribbean Europe Ghana India Iran Iranian-Kurdish Latin American Iran Iraq	Iraqi-Kurdistan Morocco Middle Eastern Nigeria North American Pakistan Palestine Russia Somalia UK Yemen Zimbabwe	^31^ ^,^ ^35^^,^ ^38^^,^ ^40^^,^ ^41^^,^ ^44^^,^ ^47^^,^ ^49^^,^ ^50^
	**Length of detention**	1 day—2 years 8 months		^34^ ^,^ ^37^^,^ ^39^^,^ ^44^^,^ ^46^^,^ ^50^
	**Numbers of staff/other stakeholders**	118 N/S = 5 articles		^17^ ^,^ ^33^^,^ ^34^^,^ ^36^^,^ ^38^^,^ ^40^^,^ ^43^^,^ ^46–49^^,^ ^51^^,^ ^54^

N/S, not specified; No., number.

### Findings of the review

Key findings are presented in tabular format (Supplementary File SE), further coded and assigned into relevant themes with associated supporting and conflicting references (Table 2).

### Basic needs

#### Physiological needs

Twenty articles examined the physical environment.^23^^,^^39–57^ Although food provision was seen positively in four articles,^39^^,^^40^^,^^46^^,^^53^ the majority of articles found detainee distress and hunger from subpar quality food, strict mealtimes, and lack of dietary choice.^41–48^^,^^54^^,^^57^ One retrospective study of four pregnant detainees found that their particular appetites and pregnancy-associated sickness was not accommodated for within the regime.^42^ Another study of six IRCs found anxiety in those with dietary requirements e.g. fear of meat in vegetarian options or non-Halal meat.^56^

Cleanliness, ventilation, and inadequate clothing provision were issues identified in four articles,^23^^,^^45^^,^^51^^,^^56^ though conditions varied across IRCs.^46^^,^^54^^,^^56^ Environmental factors further disrupted detainees’ physiological comfort: in three articles, detainees were unable to control room temperature, needing to request blankets^47^ or endure broken ventilation and non-opening windows.^46^^,^^56^ Sleep disruption was reported in nine articles, caused by noise levels including hearing planes overhead, shared rooms, staff intrusions for monitoring, and night-time admissions or transfers.^23^^,^^46–50^^,^^55–57^

Lack or loss of possessions was reported in four articles.^49^^,^^55–57^ This resulted from the sudden nature of detention (e.g. being detained at home, work, straight from prison, or during routine Home Office reporting) which prevented detainees from collecting personal belongings such as clothing and medications, or even seeing family.^55^ Additional losses occurred during transfers between facilities or searches, with detainees reporting food in rooms being confiscated.^49^^,^^56^^,^^57^ Together, these disruptions reflect detainees’ loss of control over basic needs and the immediate environment, contributing to both physiological and psychological distress.

#### Safety needs

Detainee safety was explored in 21 articles.^23^^,^^39–47^^,^^49–52^^,^^54–60^ Heightened stress levels through a reduced sense of security from physical or emotional abuse from staff or other detainees was reported in seven articles.^23^^,^^41^^,^^47^^,^^49^^,^^52^^,^^56^^,^^57^ Threats of abuse or discriminatory attitudes, including homophobia, sexism, and racism were evidenced in seven articles.^23^^,^^39^^,^^45^^,^^47^^,^^52^^,^^56^^,^^59^ Furthermore, seven articles found detainees distressed from witnessing, or awareness of, other detainees depression, self-harm or suicide attempts.^41^^,^^43^^,^^51^^,^^52^^,^^55^^,^^57^^,^^58^ Distressing incidents included but was not limited to riots, fires, ‘dirty’ protests, and hunger strikes as well as witnessing the indefinite detention of other detainees.^56^^,^^57^ One preliminary study found 20.8% of detainees self-reported safety as an unmet need^39^ whilst an inspection of Derwentside IRC found 45% of detainees reported having felt unsafe within the centre.^54^

Neglect of physical and mental health needs was reported through detainee’s experiences of being ignored or disbelieved about their ailments,^41^^,^^43^^,^^56^^,^^60^ delays in mental health referrals,^42^^,^^44^ staff shortages,^23^^,^^41^^,^^44^ and the transient nature of detention impeding continuity of care.^23^^,^^44^^,^^46^ Continuity of care was also impacted by rapid releases and poor communication between transfers, with three articles finding former detainees being left homeless.^46^^,^^49^^,^^57^ A study of eight IRCs identified that rapid release or removal hindered access to legal support and the ability of mental health staff to implement coping strategies and link with relevant support systems.^23^ Use of separation units in three articles was found to be detrimental to sleep, increasing isolation and paranoia and, in Derwentside’s HMIP inspection, was found to be used inappropriately.^23^^,^^54^^,^^56^

Access to healthcare was seen positively in both HMIP IRC inspections with use of screening, interpreting services, and access to dentists and pharmacies.^46^^,^^54^ However, despite this access, they also reported poorly organized care and poor communication amongst staff pertaining to those identified at risk of self-harm or suicide. For example, HMIP’s inspection of Tinsley House found that one detainee, classed as a ‘Level 3 adult at risk’ (defined as someone for whom detention is likely to cause harm^61^), did not receive a vulnerable adult care plan until around 4 months into their detention, after being discovered researching methods of suicide.^46^

Three articles found that medication being confiscated on arrival led to a temporary discontinuation of vital treatments or mistakes, notably HIV medication and transition hormones for transgender detainees, causing significant mental distress.^41^^,^^52^^,^^58^ HMIP’s inspection of Tinsley House reported easy access to healthcare, including drug and alcohol teams. However, no vapes were available, therefore those that vaped either had to give up or start smoking.^46^

Barriers to accessing legal support included geographical distance from legal representatives compounded by frequent inter-detention transfers^49^^,^^54^^,^^55^ and poor communication around detention status including rapid removal decisions with less than 72 h’ notice and/or occurring at night.^41^^,^^46^^,^^51^^,^^57^ Poor removal communication also occurred in several articles where detainees reported multiple failed removal attempts, some boarding planes and then returning to the IRC with no clear explanation.^49^^,^^57^ Additional obstacles included a lack of translation services,^43^^,^^44^^,^^56^ shortages in working computers^46^^,^^50^ or internet restrictions limiting access to key evidentiary sources such as LGBTQ+ websites.^50^^,^^52^

### Psychological needs

#### Belongingness

Twenty articles reported on factors affecting detainees’ sense of belongingness.^23^^,^^39^^,^^41–43^^,^^45–57^^,^^59^^,^^62^ Belongingness was conceptualized as the extent to which detainees experience social connectedness, cultural affirmation, and recognition of their social and parental identities within and beyond the detention environment.

Inadequate provision of diversity and inclusion adjustments was evident in the limited access to or use of translated information or interpreters.^41^^,^^43–46^^,^^54^^,^^56^ This related to immigration paperwork, healthcare provision, and night-time arrivals with some detainees reporting they didn’t know where they were or why. Other issues related to gender such as limited access to sanitary products,^51^ and in one IRC, the storing of halal and non-halal meat together.^46^

Distress and anxiety resulted from detainee separation from their families, especially children, challenging their identity as parents.^56^ Detention posed childcare difficulties and feelings of a lack of consideration for personal circumstances e.g. one detainee had a child in hospital whilst they were in detention.^55^ This resulted in feelings of guilt or shame and, in some cases, negative consequences on the children themselves with transfer into social care services or poor behaviour development and educational attainment.^55–57^

Social isolation between detainees, and detainees and staff, largely stemmed from language barriers,^47^^,^^56^^,^^57^ transiency of detention,^23^^,^^56^^,^^57^ and negative staff interactions or inability of staff to provide adequate emotional support.^23^^,^^43^^,^^45^^,^^54^^,^^59^ One large-scale cross-sectional study found 60% of detainees spent most of their time alone.^45^ In Derwentside IRC, 9 months after opening, 95% of detainees reported to have not had a visit since arriving.^54^ Similarly, another smaller study found 62.4% of detainees self-reported an unmet need for company.^39^ A buddy system was documented in one article, to link current detainees with new arrivals, although this was found to be emotionally challenging for both parties involved.^56^ This isolation extended into impeded communication with the ‘outside world’ through geographical and consequently financial challenges as well as practical constraints.^56^^,^^57^ These included visitor restrictions,^46^^,^^50^ lack of privacy for conversations,^47^^,^^52^ and phone and social media restrictions,^46^^,^^47^^,^^50^^,^^54^ which negatively impacted both social and professional connections. Impaired social skills and relationship difficulties were also shown to persist even after release in one large-scale ethnographic study of FNOs.^57^

Eight articles reported detainee coping strategies for detention comprised of faith and philosophy,^23^^,^^43^^,^^47^^,^^50^^,^^53^^,^^56^^,^^57^ interpersonal relationships,^23^^,^^43^^,^^47^ and advocacy.^43^ Conversely, staff in one study disclosed emotional desensitization as a coping mechanism to protect their own mental wellbeing.^23^ In a similar manner, a mutual mistrust between staff and detainees was found in three other articles.^42^^,^^56^^,^^62^ In an IRC, recently converted from a former prison, some staff members still regarded themselves as prison officers whereas where a more positive culture was found, staff referred to detainees as ‘residents’ and themselves as ‘care workers’.^56^

#### Esteem

The enforced regime inside IRCs produced feelings of a loss of agency as a detainee’s environment, activities, and time were predetermined and outside of their control.^23^^,^^47^^,^^53^ Twenty articles examined conditions affecting detainee’s esteem.^23^^,^^41–43^^,^^45–48^^,^^50–60^^,^^62^ One study, spanning eight IRCs, found that detainees in some centres were being locked in their rooms at night, and although acknowledging that restrictions varied across IRCs, it did not clarify whether any IRCs did not use locks.^23^ Feelings of dehumanization appeared in four articles.^47^^,^^55^^,^^56^^,^^60^ Detainees expressed feeling like cows or sheep,^56^ or even ‘chickens in a slaughterhouse’.^60^

Detainees expressed feelings of powerlessness induced by the hierarchy of power,^41^^,^^43^^,^^47^^,^^57^^,^^62^ with some staff exhibiting controlling behaviour and incidents being met with sanctions rather than identifying the root cause of distress.^41^ Similarly, use of strikes and privileges was found in another article inducing feelings of passivity.^57^ This passivity, compounded by feelings of hopelessness and fatigue, caused some detainees to feel like they were either zombies or dead.^57^ As a consequence, detainees in two articles expressed a reluctance to seek help or help others receiving unjust treatment due to perceived sanctions and relegations from a position of inferiority.^48^^,^^62^

Lack of privacy, with random searches and camera surveillance, was a concern identified in seven articles contributing to feelings of disrespect and emotional distress.^41^^,^^42^^,^^46^^,^^47^^,^^50–52^^,^^57^ Five articles found incidences of staff entering detainees’ rooms unannounced, including when detainees were dressing or using the toilet.^42^^,^^46^^,^^47^^,^^51^^,^^52^ Breaches of privacy extended to restrictions or monitoring of communications with the outside world such as private healthcare consultations.^41^^,^^42^ A qualitative study of pregnant women found an absence of privacy during intimate examinations and a study of LGBTQ+ detainees identified privacy issues as a barrier to discussing intimate issues relating to one’s immigration case.^42^^,^^52^

Institutionalization through strict regimes and the physicality of being detained caused detainees in two articles to feel criminalized, rather than humans caught in an administrative process.^47^^,^^59^ The process of entering and leaving detention involved being ‘arrested’ at home, work or whilst reporting to the Home Office,^56^ use of secure transportation vans, handcuffs,^46^^,^^50^^,^^57^^,^^60^ and even in one article restricted toilet use leading to a detainee defecating in their trousers.^57^ Feelings of criminality extended into the IRCs with prison-like environments including high walls, barbed wire, locked gates, bars on windows, and shared rooms.^56^^,^^57^^,^^60^ One study of FNOs and their family members found many felt a sense of permanent criminality with some spending longer in IRC detention than prison, or feeling publicly shamed during visitations.^55^

Some detainees were unsure of their wrongdoing,^47^^,^^50^^,^^57^ or felt unwanted or mistrusted causing insult to their perceived self-worth.^50^^,^^55^^,^^58^ Four articles found detainees felt forgotten or invisible due to lack of information around their detention status and duration,^49^^,^^55–57^ and some even dressed formally to try to maintain their own self-respect.^56^ A qualitative study adopting the social identity framework identified a sense of socio-political inequality, injustice, and stigmatization.^62^ Four other articles linked stigmatization to feelings of discrimination,^51^^,^^52^^,^^56^^,^^57^ and five articles found detainees with a loss of self-identity.^41^^,^^43^^,^^47^^,^^56^^,^^60^ One article examining dignity in detention found many issues persisted even after release into the UK community.^60^ These included mental health conditions, loss of self-confidence and self-esteem, and some lived in fear of re-detention with ongoing Home Office reporting.

### Self-actualization

Features of detention relevant to self-actualization were reported upon in 13 articles.^23^^,^^39^^,^^43^^,^^45–48^^,^^51^^,^^53^^,^^55–57^^,^^60^ Temporal uncertainty of the outcome of detainees’ immigration cases, and subsequent detention duration, engendered a sense of limbo.^48^^,^^51^^,^^53^ This ambiguity restricted their ability and motivation to future plan, prohibiting pursuit of long-term development.^23^^,^^49^^,^^56^^,^^57^^,^^60^ The lack of release date was directly linked to feeling depressed for detainees in one article.^55^ Whereas detainees in two articles described preferring prison to immigration detention as their prison sentences and specified timeframes made them feel respected and allowed them to imagine and plan for their futures.^57^^,^^60^

Three articles described detainees’ perceived loss of purpose or meaning in life.^23^^,^^43^^,^^53^ Detainees in one study reported feeling lost in time and an inability to concentrate due to feelings of anxiety causing anhedonia.^56^ Some reported being unable to imagine or plan a short-term day-to-day future, as well as long-term.^57^ In two articles, even those that wanted to leave and volunteer for removal felt stuck due to administrative delays and errors occurring impeding their removal.^55^^,^^56^

Physical barriers to self-actualization arose from limited educational opportunities. Specifically, the gym, library or English lessons were often restricted by time, resources and availability of trained facilitators.^23^^,^^39^^,^^46^ One study of six IRCs found detainees felt boredom from the monotony of their daily routines, limited weekend activities or perceived meaningless activities.^56^ Similar to the pay of UK prisoners,^63^ Tinsley House IRC was reported to provide paid work for detainees at a rate of £1 per hour, between £6 and £9 less than the UK’s National Minimum Wage.^46^^,^^64^

### Quality of the evidence

Critical appraisal was undertaken for 13 qualitative studies (and the qualitative component of 5 mixed methods studies), with most (15/18) scoring 70% or more. Three studies included a reflexivity statement,^47^^,^^55^^,^^56^ and over a third (7/18) reported ethical approval by a designated body.^42^^,^^43^^,^^47^^,^^48^^,^^55^^,^^58^^,^^62^ Although some reports did not explicitly name an ethics committee or approval reference, there was evidence of multiple safeguards indicating that ethical principles were applied in practice such as voluntary participation, anonymization of data, linguistic accessibility, and the cautious handling of sensitive topics. Two prevalence studies and one cross-sectional study underwent quantitative critical appraisal (and the quantitative components of five mixed methods studies), employing surveys as their quantitative methods. Scores ranged between 50% and 100%, with a mean score of 81%.

Of five mixed methods articles, one was high quality,^58^ three mixed,^23^^,^^54^^,^^56^ and one low, scoring 50% or less for both qualitative and quantitative components.^46^ Two scoping reviews scored 73% and 64%, respectively, and although not a requirement of scoping reviews, lacked critical appraisal and evaluation of publication bias.^41^^,^^44^ One piece of grey literature, unable to be appraised using study tools, was classed as Level 5 evidence according to OCEBM.^38^

Overall, this review used a mixed-quality body of evidence and strengths and limitations of individual articles are included in Supplementary File SE.

## Discussion

### Main findings of this study

This review demonstrates that conditions within UK IRCs systematically undermine detainees’ mental wellbeing through failures across all levels of human need. These findings represent structural rather than individual failings. Poor food quality, limited choice, and rigid mealtimes contributed to deprivation and psychological distress,^42^^,^^47^ while sleep disruption and barriers to physical and mental healthcare exacerbated these effects.^43^^,^^45^ Safety concerns were also pervasive, with reports of violence, self-harm, and lack of privacy fostering fear and emotional vulnerability.^23^^,^^49^^,^^52^ Inadequate planning for transfer or release often left detainees without continuity of care or housing support.^46^ These findings are consistent with the Shaw Review’s conclusion that detention compounds vulnerability and can itself deteriorate mental wellbeing when safeguards are weak.^9^ They also align with the Brook House Inquiry’s determination that harm was structurally enabled by institutional culture and inadequate oversight.^15^

Barriers to communication strained personal and professional relationships, while defective detainee-staff relationships induced feelings of isolation.^48^^,^^59^ Socio-cultural identity was frequently undermined by racial and gender discrimination and inadequate provisions for diversity and inclusion.^46^^,^^52^ Limited educational or meaningful activity contributed to stagnation and loss of purpose.^45^^,^^46^ The indefinite nature of detention and absence of clear communication about legal case progress eroded detainee’s sense of self-worth and sense of meaning.^53^ This diminished motivation for self-actualization, even if opportunities, albeit limited, were offered.

Although the included studies varied in quality, most provided rich qualitative data through in-depth interviews and ethnography. However, convenience or snowball sampling introduced potential bias, and grey literature often lacked reflexivity, particularly where researchers had direct relationships with participants such as advocacy-adjacent research roles. This reflects well-documented challenges of conducting research within the UK immigration detention system, which involves vulnerable and transient populations, restricted institutional access, language barriers, and ethical and safeguarding considerations that complicate informed consent, data reliability, and researcher independence.^9^^,^^56^

### What is already known on this topic

Existing literature indicates that being detained is associated with adverse mental health outcomes and the conditions of detention are a probable contributing factor. International longitudinal studies have shown that prolonged time in detention correlates with worsening severity of psychological symptoms with significant improvements exhibited at follow-ups of released detainees.^21^^,^^22^ Gerlach’s qualitative work on women in UK immigration detention conceptualizes detention as an erosion of dignity, arguing that institutional practices, carceral architecture, and staff–detainee interactions systematically undermine autonomy and self-worth.^65^ Her analysis highlights detention as a continuum of harm extending beyond release, reinforcing how structural conditions produce lasting psychosocial consequences.

To date there has been a dearth of UK-based tertiary research on mental wellbeing relating to IRCs. However, grey literature and primary research makes apparent the complex psycho-social interplay between immigration detention and mental health and wellbeing.^66^^,^^67^ These relate not only to the detainee’s own trauma and vulnerabilities, such as racial or gender persecution, human trafficking or mental and physical health problems, but to the detention environment and the physical and psychological stress it induces.^67^ The ways in which detainees spend their time in detention can aid in providing feelings of hope or connection, such as through routine, self-care and religious activities, whilst others find their sense of purpose and control through working on their legal cases.^68^ Conversely, detention itself has been shown to be detrimental to some detainees’ ability and motivation to occupy themselves.^68^

### What this study adds

This is among the first evidence syntheses focussed solely on IRCs in the UK. It identifies the physical and psychological conditions created by detention and how they act to the detriment of detainee’s mental wellbeing and subsequently, mental health. By mapping detainee experiences onto Maslow’s dimensions, this review provides a structured framework that operationalizes concerns raised by government reports,^9^^,^^15^ as well as academic research.^65^ Together, these sources indicate that psychological harm within IRCs is structurally reproduced through uncertainty, restricted agency, institutional opacity, and carceral design.

Findings within this review provide a foundation for practical improvements to IRCs. Deficiencies in basic living conditions such as food, sleep, hygiene, and healthcare provision were associated with emotional suffering, disempowerment, and psychological distress. Potential improvements include food quality with appropriate cultural and health-related dietary options, access to private, single-occupancy rooms, and enhanced standards of cleanliness, clothing, and sanitation. Although some IRCs have introduced cultural kitchens, overall provision remains poor.^67^

Healthcare within IRCs shows significant shortcomings, particularly regarding continuity of care during transfer, removal, or release. This highlights the need for stronger policies and procedures to ensure uninterrupted, culturally competent care. Improved communication and coordination amongst all professionals involved, and most importantly with detainees, must be addressed to support this. Poor communication between agencies, coupled with limited transparency from the Home Office, reflects a wider institutional culture of opacity that has drawn scrutiny since the Windrush Scandal.^69^^,^^70^ Strengthening communication channels—both with detainees and between agencies—is therefore not merely an operational reform but a moral imperative for rebuilding public trust and institutional accountability.

Feelings of insecurity and unfulfilled needs were particularly acute for vulnerable detainees (pregnant women, LGBTQ+, trauma survivors, those with chronic health needs). Similar concerns raised by other studies and healthcare organizations,^66^^,^^71^ indicate the need for stronger safeguarding measures and stricter scrutiny of detention decisions to assess necessity and suitability.

The psychological toll of temporal uncertainty was a recurring theme. The indefinite nature of detention, lack of clear communication about its rationale or duration, and inability to plan for the future incurred feelings of hopelessness and discrimination. Many detainees experienced social isolation, a diminished sense of identity and self-worth, and, in some cases, further withdrawal from family and friends due to shame. Notably, the institutional environment often mirrored carceral settings, fostering feelings of criminalization, stigma, and dehumanization. Reducing the physical and social restrictions of IRCs, including their design and operational culture, may help to mitigate some of these feelings. While staff training in cultural competence and empathy may help restore detainees’ sense of agency, respect, and purpose.

The UK remains the only country in Europe without a statutory time limit on immigration detention, despite repeated criticism from oversight bodies and human rights organizations.^26^^,^^72^ The implementation of time limits, together with transparent communication about case progression, would not only reduce distress but also align UK practice with international human rights standards.^73^ In the meantime, with around 32% of detainees in the year ending June 2025 spending between 1 and 6 months in detention, and 2% spending between 6 months and over 4 years in detention, targeted strategies are urgently needed to support those in prolonged detention and prevent the adverse effects of institutionalization.^74^ The Shaw report’s recommendation to reduce reliance on detention and expand alternatives remains highly relevant in light of the findings that prolonged uncertainty exacerbates psychological harm.^9^

These findings must be viewed within the broader context of evolving UK immigration policy, which has increasingly prioritized detention as a mechanism of border control. The Illegal Migration Act (2023), which supersedes the data analysed within this review, grants the Home Office extended powers to detain indefinitely and limits judicial oversight, effectively normalizing prolonged detention.^13^ Such policy developments may exacerbate the psychological harms identified in this review, particularly those arising from uncertainty, loss of agency, and restricted access to legal recourse.

Findings suggest varying conditions across different IRCs which is supported by current literature.^75^ HMIP reports worsening conditions despite repeated recommendations at previous inspections.^76^ Nonetheless, the government plans to expand the detention estate through the re-opening of Campsfield House and Haslar; IRCs previously closed following concerns of abuse and neglect.^77^ The repeated closure and opening of IRCs has created a volatile system characterized by fluctuating capacity, inconsistent standards, and potential overcrowding.^78^ Such instability risks amplifying the poor conditions and psychosocial stressors documented here, particularly if expansion proceeds without robust safeguards and independent oversight.

While some centres may demonstrate better practices, this review’s focus was on identifying the factors harming mental wellbeing. Future research could explore effective compassionate practices. Ultimately, it raises a broader question of whether detention is even necessary at all. Thought must be given as to what the purpose of administrative (not criminal) detention really is, who it is protecting, and whether current facilities, processes and subsequent severe restrictions to detainees’ daily lives align with this purpose. Community-based alternatives, such as social housing, may address many of the issues identified in this review whilst potentially offering cost-effective solutions for the government.^79^

At a time when the UK government is expanding the detention estate amid ongoing debates about migration and human rights, the evidence presented here highlights an important consideration: whether immigration detention can remain an administrative process governed by law and humanity, or risks further adopting features of a punitive system.

### Limitations of this study

Similar to any review, this review was susceptible to researcher bias and human error. The broad scope may have led to a limited depth of analysis and oversimplification of complex and diverse experiences. Differences between individual IRCs were not explicitly examined, despite restrictions likely varying across centres. Many articles did not specify which IRC participants were held in. Furthermore, four of the IRCs (The Verne, Morton Hall, Campsfield House, Dover) have since closed or been re-purposed, potentially limiting applicability of this review. Similarly, applicability is restricted due to some study timeframes being superseded by legislative changes and policy reforms.

## Conclusions

This review, through focussing on the aspects of detention and structures of institutions that have engendered mental wellbeing issues amongst detainees, provides specific guidance for improvements and foundations for future research in this area. Deficiencies in all dimensions, of Maslow’s (1943) Hierarchy of Human Needs, were shown to negatively influence detainee mental wellbeing, leading to emotional suffering, disempowerment, and feelings of dehumanization and criminality. Improving conditions including staff satisfaction within IRCs may yield dual benefits through safeguarding detainee’s mental wellbeing and concurrently enhancing overall management effectiveness. Establishing time limits on detention and communicating these effectively would likely enable detainees to better psychologically process and cope with their immigration status. Careful consideration should also be given to any community-based, or alternative, settings chosen for immigration administration purposes, while reviewing the criteria and standards of care applied during detention.

## Supplementary Material

Supplementary_material_fdag044

## Data Availability

The data underlying this article are available in the article and in its online supplementary material.

## Notes

**Niamh Gallagher**, Clinical Skills Tutor

**Tristan Price**, Associate Professor of Clinical Education

**Matthew Griggs**, Programme Lead MSc Expedition and Marine Medicine and Lecturer MSc Global Health

## References

[1] Migration Observatory . *Immigration Detention in the UK*. Oxford: University of Oxford COMPAS (Centre on Migration, Policy and Society), 2022.

[2] McGuinness T, Gower M, Sturge G et al. *Immigration Detention in the UK: An Overview*. House of Commons Library. London: HM Parliament, 2018.

[3] Home Office . *Immigration System Statistics, Year Ending June 2023: How Many People Are Detained or Returned?* London: UK Government, 2023.

[4] HMIP . *Report on an Unannounced Inspection of Brook House Immigration Removal Centre*. London: HM Inspectorate of Prisons, 2022.

[5] Chambers Hannah, Plotkin Ariel, Hanley Idel. Harmed not heard. London: Medical Justice, 2022.

[6] Bail for Immigration Detainees . Written evidence submitted by Bail for Immigration Detainees. In: Committee HA (ed.), Immigration Inquiry. UK Parliament, 2017.

[7] Taylor B, Girvan A, Matthews L. A History of Immigration Detention in the UK (1914-2018). London: University of East Anglia; Refugee History; Right to Remain, 2018.

[8] Jerome Phelps A, Ben du Preez, Hamid, Kuka, Magali Carette, Matthew, Sharif and Souleymane. The State of Detention. London: Detention Action; 2014.

[9] Shaw S . *Review into the Welfare in Detention of Vulnerable Persons*. Parliament, Department SoSfH, 2016.

[10] Taylor B, Girvan A, Matthews L. *A History of Immigration Detention in the UK (1914–2018)*. Right to Remain, 2018.

[11] Joint Select Committee on Human Rights . *Windrush Generation Detention: Government Response to the Committee’s Sixth Report of Session 2017–19*. London: HM Parliament, 2019.

[12] Home Office . *Immigration Statistics Quarterly Release, how Many People Are Detained or Returned?* London: Home Office, 2019.

[13] Illegal Migration Act. London: HMSO; 2023. [Available from: https://www.legislation.gov.uk/ukpga/2023/37/contents].

[14] Joint Committee on Human Rights. Legislative Scrutiny: Illegal Migration Bill. London: HM Parliament, 2023.

[15] Home Office . *Brook House Inquiry*. London: UK Government, 2023.

[16] Robjant K, Hassan R, Katona C. Mental health implications of detaining asylum seekers: systematic review. Br J Psychiatry 2009;194:306–12. 10.1192/bjp.bp.108.053223 19336779

[17] Procter NG, De Leo D, Newman L. Suicide and self-harm prevention for people in immigration detention. Med J Aust 2013;199:730–2. 10.5694/mja13.10804 24329621

[18] Silove D, Austin P, Steel Z. No refuge from terror: the impact of detention on the mental health of trauma-affected refugees seeking asylum in Australia. Transcult Psychiatry 2007;44:359–93. 10.1177/1363461507081637 17938152

[19] Bosworth M, Kellezi B. Quality of life in detention: results from MQLD questionnaire data collected in IRC Campsfield House, IRC Yarl’s Wood, IRC Colnbrook, and IRC Dover, September 2013–August 2014. SSRN Electron J 2015. 10.2139/ssrn.2569667

[20] Robjant K, Robbins I, Senior V. Psychological distress amongst immigration detainees: A cross-sectional questionnaire study. Br J Clin Psychol 2009;48:275–86. 10.1348/014466508X397007 19200410

[21] Steel Z, Silove D, Brooks R et al. Impact of immigration detention and temporary protection on the mental health of refugees. Br J Psychiatry 2006;188:58–64. 10.1192/bjp.bp.104.007864 16388071

[22] Keller AS, Rosenfeld B, Trinh-Shevrin C et al. Mental health of detained asylum seekers. Lancet 2003;362:1721–3. 10.1016/S0140-6736(03)14846-5 14643122

[23] Durcan G, Stubbs J, Boardman J. Immigration Removal Centres in England: A mental health needs analysis. London: Centre for Mental Health, 2017.

[24] Home Office . *Detention: General Instructions*, 6.0 edn, 2025.

[25] UNHCR . Detention Guidelines. Switzerland: UNHCR, 2012.

[26] HMIP . *The Effectiveness and Impact of Immigration Detention Casework: A Joint Thematic Review*. London: HM Inspectorate of Prisons, 2012.

[27] Maslow AH . A theory of human motivation. Psychol Rev 1943;50:370–96. 10.1037/h0054346

[28] JBI . Chapter 11: Scoping Reviews 2022. https://jbi-global-wiki.refined.site/space/MANUAL/4687342/Chapter+11%3A+Scoping+reviews.

[29] JBI . Chapter 2: systematic reviews of qualitative evidence [Internet]. In: Aromataris E, Lockwood C, Porritt K, Pilla B, Jordan Z (eds.), *JBI Manual for Evidence Synthesis*. Australia: Joanna Briggs Institute, 2022. https://jbi-global-wiki.refined.site/space/MANUAL/4688637/Chapter+2%3A+Systematic+reviews+of+qualitative+evidence (27 August 2023, date last accessed).

[30] Mishra JN . *Cross-Cultural Study of Maslow's Need Theory of Motivation*. New Jersey Institute of Technology, 1985.

[31] Henwood BF, Derejko KS, Couture J et al. Maslow and mental health recovery: a comparative study of homeless programs for adults with serious mental illness. Adm Policy Ment Health 2015;42:220–8. 10.1007/s10488-014-0542-8 24518968 PMC4130906

[32] Benson S, Dundis S. Understanding and motivating health care employees: integrating Maslow’s hierarchy of needs, training and technology. J Nurs Manag 2003;11:315–20. 10.1046/j.1365-2834.2003.00409.x 12930537

[33] Schriger SH, Binagwaho A, Keetile M et al. Hierarchy of qualities in global health partnerships: a path towards equity and sustainability. BMJ Glob Health. 2021;6. 10.1136/bmjgh-2021-007132 PMC8718486 34969686

[34] JBI . JBI critical appraisal checklist for qualitative research. In:Francis E (ed.), Australia: Joanna Briggs Institute, 2022.

[35] JBI . Chapter 5: systematic reviews of prevalence and incidence. In:Munn ZMS, Lisy K, Riitano D, Tufanaru C (eds.), *JBI Manual for Evidence Synthesis*. Australia: Joanna Briggs Institute, 2020.

[36] JBI . Chapter 7: systematic reviews of etiology and risk. In:Munn ZMS, Tufanaru C, Aromataris E et al. (eds.), *JBI Manual for Evidence Synthesis*. Australia: Joanna Briggs Institute, 2020.

[37] Aromataris EFR, Godfrey C, Holly C et al. Summarizing systematic reviews: methodological development, conduct and reporting of an umbrella review approach. Int J Evid Based Healthc 2015;13:132–40. 10.1097/XEB.0000000000000055 26360830

[38] OCEBM Levels of Evidence Working Group . *The Oxford 2011 Levels of Evidence*. Oxford: Oxford Centre for Evidence-Based Medicine, 2011.

[39] Sen P, Arugnanaseelan J, Connell E et al. Mental health morbidity among people subject to immigration detention in the UK: a feasibility study. Epidemiol Psychiatr Sci 2018;27:628–37. 10.1017/S2045796017000269 28637536 PMC6999003

[40] Sen P, Crowley G, Moro C et al. Mental health in immigration detention: A comparison of foreign national ex-prisoners and other detainees. Crim BehavMent Heal 2021;31:275–87. 10.1002/cbm.2207 34392577

[41] Van Hout M-C, Lungu-Byrne C, Germain J. Migrant health situation when detained in European immigration detention centres: A synthesis of extant qualitative literature. Int J Prison Health 2020;16:221–36. 10.1108/IJPH-12-2019-0074 33634662

[42] Arshad F, Haith-Cooper M, Palloti P. The experiences of pregnant migrant women in detention: a qualitative study. Br J Midwifery 2018;26:591–6. 10.12968/bjom.2018.26.9.591

[43] Hollis J . The psychosocial experience of UK immigration detention. Int J Migr Health Social Care 2019;15:76–89. 10.1108/IJMHSC-04-2018-0024

[44] Lungu-Byrne C, Germain J, Plugge E et al. Contemporary migrant health experience and unique health care needs in European prisons and immigration detention settings. Int J Forensic Ment Health 2021;20:80–99. 10.1080/14999013.2020.1821129

[45] Bosworth M, Gerlach A. *Quality of Life in Detention Results from MQLD Questionnaire Data Collected in Gatwick IRC (Brook House and Tinsley House), Heathrow IRC (Colnbrook and Harmondsworth), Yarl’s Wood IRC, Morton Hall IRC, and Dungavel IRC: July 4–September 20*. Centre for Criminology University of Oxford, 2019, 2020.

[46] HMIP . *Report on an Unannounced Inspection of Tinsley House Immigration Removal Centre*. London: HM Inspectorate of Prisons, 2023.

[47] Gallagher A . The impact of immigration detention on the mental health of adults—section B: “I Went to Hell and Back”. In: *Investigating how Psychosocial Processes of Immigration Detention Affect Ex-detainees’ Mental Health*. repository.canterbury.ac.uk: Canterbury Christ Church University, 2017.

[48] Godshaw D . Becoming an immigrant? Border harms and “British” men with previous convictions in British immigration removal centers. Crit Criminol 2020;28:225–41. 10.1007/s10612-020-09524-2

[49] Griffiths MBE . Out of time: The temporal uncertainties of refused asylum seekers and immigration detainees. J Ethn Migr Stud 2014;40:1991–2009. 10.1080/1369183X.2014.907737

[50] Right to Remain . Immigration detention zine: a rough guide for people at risk and their supporters. In: Rosie (ed.). London: Right to Remain, 2019.

[51] Canning V . Reimagining Refugee Rights: Addressing Asylum Harms in Britain, Denmark and Sweden. 978-1-5272-3092-7 I, 2019.

[52] UKLIG and Stonewall . *No Safe Refuge: Experiences of LGBT Asylum Seekers in Detention*. London: UK Lesbian & Gay Immigration Group, 2016.

[53] Turnbull S . ‘Stuck in the middle’: waiting and uncertainty in immigration detention. Time Soc 2016;25:61–79. 10.1177/0961463X15604518

[54] HMIP . *Report on an Unannounced Inspection of Derwentside Immigration Removal Centre*. London: HM Inspectorate of Prisons, 2022.

[55] Hasselberg I . *Enduring Uncertainty: Deportation*. Punishment and Everyday Life: Berghahn Books, 2016.

[56] Bosworth M . *Inside Immigration Detention*, Online edn. Oxford: Oxford University Press, 2014.

[57] Griffiths M . Living with uncertainty: indefinite immigration detention. Journal of Legal Anthropology 2013;1:263–86. 10.3167/jla.2013.010301

[58] Zimmerman SE, Chatty D, Nørredam ML. Health needs and access to care in immigration detention: perceptions of former detainees. Int J Migr Health Soc Care 2012;8:180–5. 10.1108/17479891211287094

[59] Lawlor D, Sher M. The use of detention as a defence against intolerable social anxiety towards asylum seekers. Socioanalysis 2016;18:40–52.

[60] Gerlach A . Women’s experiences of indignity in immigration detention and beyond. Incarceration 2022;3:26326663221103437. 10.1177/26326663221103437

[61] Home Office. Adults at risk in immigration detention (Accessible). London: UK Government, 2024. [Updated 27/08/2025. Available from: https://www.gov.uk/government/publications/adults-at-risk-in-immigration-detention/adults-at-risk-in-immigration-detention-accessible-version#purpose-and-background].

[62] Këllezi B, Wakefield J, Bowe M et al. Healthcare provision inside immigration removal centres: a social identity analysis of trust, legitimacy and disengagement. Appl Psychol Health Well Being 2021;13:578–601. 10.1111/aphw.12263 33755329

[63] HM Prison Service . *Prisoner’s Pay*. London: UK Government, 2020.

[64] UK Government . *The National Minimum Wage and Living Wage*. London: UK Government, 2023. https://www.gov.uk/national-minimum-wage/who-gets-the-minimum-wage

[65] Gerlach A . *Dignity, Women, and Immigration Detention*, 1st edn. London: Routledge, 2022.

[66] BMA . *Locked up, Locked out: Health and Human Rights in Immigration Detention*. London: British Medical Association, 2017.

[67] Modha B . Detainees within immigration removal centres: a highly unique cohort of dental patients. J Health Equity 2025;2:2461012. 10.1080/29944694.2025.2461012

[68] Denny M, Twinley R. “It’s a fight with your mind”: experiences and meaning of occupation among men detained in immigration removal centres within the United Kingdom. J Occup Sci 2024;31:544–58. 10.1080/14427591.2024.2394628

[69] Clyne Rhys, Sachin S. Home truths: Cultural and institutional problems at the Home Office. London: Institute for Government, 2023.

[70] Home Affairs Committee . Immigration Detention. London: HM Parliament, 2019.

[71] Harvey L . *LGBTQI+ people’s Experiences of Immigration Detention: A Pilot Study*. London: Rainbow Migration, University of Brighton, 2023.

[72] EPRS . *The Return Directive 2008/115/EC: European Implementation Assessment*. European Parliament: European Parliamentary Research Service, 2020.

[73] UN High Commissioner for Refugees (UNHCR) . Guidelines on the Applicable Criteria and Standards Relating to the Detention of Asylum-Seekers and Alternatives to Detention. Switzerland: UNHCR, 2012.

[74] Home Office . *Summary of Latest Statistics*. London: UK Government, 2025.

[75] Migration Observatory. Immigration Detention in the UK. Oxford: University of Oxford COMPAS (Centre on Migration, Policy and Society), 2024. [Available from: https://migrationobservatory.ox.ac.uk/resources/briefings/immigration-detention-in-the-uk/#:∼:text=Official%20investigations%20have%20found%20that,poor%20care%2C%20safety%20and%20cleanliness].

[76] HMIP . *HM Chief Inspector of Prisons for England and Wales Annual Report 2024–25*. London: HM Inspectorate of Prisons, 2025.

[77] Medical Justice . ‘A Secret Punishment’—the Misuse of Segregation in Immigration Detention. London: Medical Justice, 2015.

[78] Independent Monitoring Board (IMB) . *National Annual Report 2024: Adult Prisons, Young Offender Institutions and Immigration Detention*. London: IMB, 2025.

[79] Kevin M, Meena V, Kunal S. *An Economic Analysis of Alternatives to Long-Term Detention*. Detention Action, 2012.

