# Supplementary material for: How conditions within immigration removal centres in the United Kingdom affect the mental wellbeing of detainees: a scoping review
Source: J Public Health (Oxf). 2026 Jun 26;48(3):767–79. doi: 10.1093/pubmed/fdag044 (PMC13531703; doi:10.1093/pubmed/fdag044)
Supplement: Supplementary file 1 [file supplementary_material_fdag044.zip › Supplementary File A_fdag044(1).docx]

Supplementary File A: Table of immigration detention facilities in the UK as of 30 August 2023

| **Detention Centre** | **Type of Centre** | **Population Detained** | **Maximum capacity** |
| --- | --- | --- | --- |
| Brook House, Gatwick (1) | IRC* | Men | 450 |
| Colnbrook, Middlesex (2) | IRC* | Men; Women (limit 18) | 330 |
| Derwentside, County Durham (3) | IRC* | Women | 84 |
| Dungavel House, South Lanarkshire (4) | IRC* | Men; Women | 125 |
| Harmondsworth, Middlesex (5) | IRC* | Men | 635 |
| Larne House, Antrim (6) | rSTHF^ | Men; Women | 19 |
| Manchester (6) | rSTHF^ | Men; Women | 32 |
| Swinderby (7) | rSTHF^ | Men | 39 |
| Tinsley House, Gatwick (8) | IRC* and Pre-departure accommodation | Men; Families | 162 |
| Yarl’s Wood, Bedfordshire (6) | IRC* and rSTHF^ | Men; Women | 410 |
| Notes: | *Immigration Removal Centre  ^Residential Short-Term Holding Facility | | |

References:

1.	HMIP. Report on an unannounced inspection of Brook House Immigration Removal Centre. HM Chief Inspector of Prisons; 2022.

2.	HMIP. Report on an unannounced inspection of Colnbrook Immigration Removal Centre. HM Chief Inspector of Prisons; 2022.

3.	HMIP. Report on an unannounced inspection of Derwentside Immigration Removal Centre. HM Chief Insepctor or Prisons; 2022.

4.	HMIP. Report on an unannounced inspection of Dungavel Immigration Removal Centre. HM Chief Insepctor or Prisons; 2022.

5.	HMIP. Report on a scrutiny visit to Harmondsworth immigration removal centre. HM Chief Insepctor of Prisons; 2021.

6.	HMIP. Report on an unannounced inspection of the residential short-term holding facilities at Larne House, Manchester Airport and Yarl’s Wood. HM Inspectorate of Prisons; 2021 23 - 26 August 2021.

7.	NHS. Primary Care Nurse NHS Jobs: Nottinghamshire Healthcare NHS Foundation Trust; 2022 [Available from: <https://beta.jobs.nhs.uk/candidate/jobadvert/C9186-22-0013>.

8.	HMIP. Report on an unannounced inspection of Tinsley House Immigration Removal Centre. HM Chief Inspector of Prisons; 2023.
