# Supplementary material for: How conditions within immigration removal centres in the United Kingdom affect the mental wellbeing of detainees: a scoping review
Source: J Public Health (Oxf). 2026 Jun 26;48(3):767–79. doi: 10.1093/pubmed/fdag044 (PMC13531703; doi:10.1093/pubmed/fdag044)
Supplement: Supplementary file 1 [file supplementary_material_fdag044.zip › Supplementary File B_fdag044.docx]

Supplementary File B: Exclusion Criteria

**Exclusion Criteria**

Published prior to January 2001

Not published in English and no English version available

No full-text available

Text and opinion papers and letters written by a singular author excluded due to validity and reliability concerns

No comment on conditions within the IRC

Study population did not include persons who were or had been detained in an IRC
