# Supplementary material for: How conditions within immigration removal centres in the United Kingdom affect the mental wellbeing of detainees: a scoping review
Source: J Public Health (Oxf). 2026 Jun 26;48(3):767–79. doi: 10.1093/pubmed/fdag044 (PMC13531703; doi:10.1093/pubmed/fdag044)
Supplement: Supplementary file 1 [file supplementary_material_fdag044.zip › Supplementary File C_fdag044.docx]

Supplementary File C: Full search terms and strategies

| **Subset A** | **AND** | **Subset B** | **AND** | **Subset C** |
| --- | --- | --- | --- | --- |
| Immigration  Immigrant  Refugee*  “Asylum seeker*”  “Foreign national offender*” |  | Removal  Detention |  | “Mental health”  Welfare  Wellbeing  Well-being  Psychia*  Psycho* |
| Notes: | Search constraints: Date range 1 January 2001 – 30 August 2023, with full-text available in English language  Operators: Terms within subset combined with “OR”; Complete subsets combined with “AND”  Not including thesaurus terms for each database. | | | |

Wednesday, August 30, 2023 2:01:56 PM

| **#** | **Query** | **Limiters/Expanders** | **Last Run Via** | **Results** |
| --- | --- | --- | --- | --- |
| S9 | S3 AND S4 AND S8 | Limiters - Published  Date: 20010101-  20231231 Expanders - Apply equivalent subjects Narrow by Language: english  Search modes Boolean/Phrase | Interface - EBSCOhost  Research Databases  Search Screen - Advanced  Search  Database - CINAHL Plus with  Full Text | 289 |
| S8 | S5 OR S6 OR S7 | Expanders - Apply equivalent subjects Search modes Boolean/Phrase | Interface - EBSCOhost  Research Databases  Search Screen - Advanced  Search  Database - CINAHL Plus with  Full Text | 1,164,093 |
| S7 | "mental health" OR  Welfare OR Wellbeing  OR Well-being OR  Psychia* OR Psycho* | Expanders - Apply equivalent subjects Search modes Boolean/Phrase | Interface - EBSCOhost  Research Databases  Search Screen - Advanced  Search  Database - CINAHL Plus with  Full Text | 1,164,093 |
| S6 | (MH "Psychological Well-  Being") | Expanders - Apply equivalent subjects Search modes Boolean/Phrase | Interface - EBSCOhost  Research Databases  Search Screen - Advanced  Search  Database - CINAHL Plus with  Full Text | 36,884 |
| S5 | (MH "Mental Health") | Expanders - Apply equivalent subjects Search modes Boolean/Phrase | Interface - EBSCOhost  Research Databases  Search Screen - Advanced  Search  Database - CINAHL Plus with  Full Text | 57,124 |
| S4 | removal or detention | Expanders - Apply equivalent subjects Search modes Boolean/Phrase | Interface - EBSCOhost  Research Databases  Search Screen - Advanced  Search | 48,878 |

Database - CINAHL Plus with Full Text

S3	S1 OR S2	Expanders - Apply	Interface - EBSCOhost	42,285

equivalent subjects	Research Databases

Search modes -	Search Screen - Advanced

Boolean/Phrase	Search

Database - CINAHL Plus with Full Text

S2	immigration OR	Expanders - Apply	Interface - EBSCOhost	42,285

immigrant* or refugee*	equivalent subjects	Research Databases OR "asylum seeker*" OR	Search modes -	Search Screen - Advanced

"foreign national	Boolean/Phrase	Search

offender*"	Database - CINAHL Plus with

Full Text

S1	(MH "Emigration and	Expanders - Apply	Interface - EBSCOhost	Display

Immigration")	equivalent subjects	Research Databases

Search modes -	Search Screen - Advanced

Boolean/Phrase	Search

Database - MEDLINE

Wednesday, August 30, 2023 2:13:06 PM

| **#** | **Query** | **Limiters/Expanders** | **Last Run Via** | **Results** |
| --- | --- | --- | --- | --- |
| S10 | S4 AND S5 AND S9 | Limiters - Publication  Year: 20010101-  20231231 Expanders - Apply equivalent subjects Narrow by Language: english  Search modes Boolean/Phrase | Interface - EBSCOhost  Research Databases  Search Screen - Advanced  Search  Database - Global Health | 102 |
| S9 | S6 OR S7 OR S8 | Expanders - Apply equivalent subjects Search modes Boolean/Phrase | Interface - EBSCOhost  Research Databases  Search Screen - Advanced  Search  Database - Global Health | 439,306 |
| S8 | "mental health" OR  Welfare OR Wellbeing  OR Well-being OR  Psychia* OR Psycho* | Expanders - Apply equivalent subjects Search modes Boolean/Phrase | Interface - EBSCOhost  Research Databases  Search Screen - Advanced  Search  Database - Global Health | 437,760 |
| S7 | DE "mental stress" OR  DE "psychology" OR DE  "wellness" OR DE  "psychological needs" | Expanders - Apply equivalent subjects Search modes Boolean/Phrase | Interface - EBSCOhost  Research Databases  Search Screen - Advanced  Search  Database - Global Health | 41,118 |
| S6 | DE "mental health" | Expanders - Apply equivalent subjects Search modes Boolean/Phrase | Interface - EBSCOhost  Research Databases  Search Screen - Advanced  Search  Database - Global Health | 39,277 |
| S5 | removal OR detention | Expanders - Apply equivalent subjects Search modes Boolean/Phrase | Interface - EBSCOhost  Research Databases  Search Screen - Advanced  Search  Database - Global Health | 57,948 |
| S4 | S1 OR S2 OR S3 | Expanders - Apply equivalent subjects | Interface - EBSCOhost  Research Databases  Search Screen - Advanced | 19,610 |

Search modes -	Search

Boolean/Phrase	Database - Global Health

S3	immigration OR	Expanders - Apply	Interface - EBSCOhost	19,610

immigrant* or refugee*	equivalent subjects	Research Databases OR "asylum seeker*" OR	Search modes -	Search Screen - Advanced

"foreign national	Boolean/Phrase	Search

offender*"	Database - Global Health

S2	DE "immigrants"	Expanders - Apply	Interface - EBSCOhost	8,305

equivalent subjects	Research Databases

Search modes -	Search Screen - Advanced

Boolean/Phrase	Search

Database - Global Health

S1	DE "immigration"	Expanders - Apply	Interface - EBSCOhost	1,925

equivalent subjects	Research Databases

Search modes -	Search Screen - Advanced

Boolean/Phrase	Search

Database - Global Health

Wednesday, August 30, 2023 1:59:25 PM

| **#** | **Query** | **Limiters/Expanders** | **Last Run Via** | **Results** |
| --- | --- | --- | --- | --- |
| S9 | S3 AND S4 AND S8 | Limiters - Date of  Publication: 20010101-  20231231 Expanders - Apply equivalent subjects Narrow by Language: english  Search modes Boolean/Phrase | Interface - EBSCOhost  Research Databases  Search Screen - Advanced  Search  Database - MEDLINE | 367 |
| S8 | S5 OR S6 OR S7 | Expanders - Apply equivalent subjects Search modes Boolean/Phrase | Interface - EBSCOhost  Research Databases  Search Screen - Advanced  Search  Database - MEDLINE | 3,265,985 |
| S7 | "mental health" OR  Welfare OR Wellbeing  OR Well-being OR  Psychia* OR Psycho* | Expanders - Apply equivalent subjects Search modes Boolean/Phrase | Interface - EBSCOhost  Research Databases  Search Screen - Advanced  Search  Database - MEDLINE | 3,265,985 |
| S6 | (MH "Psychological Well-  Being") | Expanders - Apply equivalent subjects Search modes Boolean/Phrase | Interface - EBSCOhost  Research Databases  Search Screen - Advanced  Search  Database - MEDLINE | 233 |
| S5 | (MH "Mental Health") | Expanders - Apply equivalent subjects Search modes Boolean/Phrase | Interface - EBSCOhost  Research Databases  Search Screen - Advanced  Search  Database - MEDLINE | 62,136 |
| S4 | removal or detention | Expanders - Apply equivalent subjects Search modes Boolean/Phrase | Interface - EBSCOhost  Research Databases  Search Screen - Advanced  Search  Database - MEDLINE | 443,622 |
| S3 | S1 OR S2 | Expanders - Apply equivalent subjects | Interface - EBSCOhost  Research Databases  Search Screen - Advanced | 84,200 |

Search modes -	Search

Boolean/Phrase	Database - MEDLINE

S2	immigration OR	Expanders - Apply	Interface - EBSCOhost	84,200

immigrant* or refugee*	equivalent subjects	Research Databases OR "asylum seeker*" OR	Search modes -	Search Screen - Advanced

"foreign national	Boolean/Phrase	Search

offender*"	Database - MEDLINE

S1	(MH "Emigration and	Expanders - Apply	Interface - EBSCOhost	26,307

Immigration")	equivalent subjects	Research Databases

Search modes -	Search Screen - Advanced

Boolean/Phrase	Search

Database - MEDLINE

Search Strategy from ProQuest

30	August 2023 14:24

# SEARCH STRATEGY

| Set No. Searched for Databases | | | Results |
| --- | --- | --- | --- |
| S1 | MAINSUBJECT.EXACT.EXPL  ODE("Immigration") | APA PsycInfo® | 27069 |
| S2 | immigration OR immigrant* OR refugee* OR "asylum seeker*" OR "foreign national offender*" | APA PsycInfo® | 53673 |
| S3 | [S1] OR [S2] | APA PsycInfo®  These databases are searched for part of your query. | 53673 |
| S4 | removal OR detention | APA PsycInfo® | 19811 |
| S5 | MAINSUBJECT.EXACT.EXPL  ODE("Well Being") OR  (MAINSUBJECT.EXACT.EXP  LODE("Psychological Needs")  OR  MAINSUBJECT.EXACT.EXPL  ODE("Psychological Stress"))  OR  MAINSUBJECT.EXACT.EXPL  ODE("Mental Health") | APA PsycInfo® | 157348 |
| S6 | "mental health" OR Welfare  OR Wellbeing OR Well-being  OR Psychia* OR Psycho* | APA PsycInfo® | 3556439 |
| S7 | [S5] OR [S6] | APA PsycInfo®  These databases are searched for part of your query. | 3559651 |
| S10 | ([S3] AND [S4] AND [S7]) AND  (la.exact("ENG") AND pd(20010101-20231231)) | APA PsycInfo®  These databases are searched for part of your query. | 437 |

Database copyright	 2023 ProQuest LLC. All rights reserved.

[Terms and Conditions](https://www.proquest.com/info/termsAndConditions) [Contact ProQuest](http://about.proquest.com/go/pqissupportcontact)

Wednesday, August 30, 2023 2:07:50 PM

| **#** | **Query** | **Limiters/Expanders** | **Last Run Via** | **Results** |
| --- | --- | --- | --- | --- |
| S11 | (S7 OR S8 OR S9) AND  (S3 AND S6 AND S10) | Limiters - Date of  Publication: 20010101-  20231231 Expanders - Apply equivalent subjects Narrow by Language: english  Search modes Boolean/Phrase | Interface - EBSCOhost  Research Databases  Search Screen - Advanced  Search  Database - SocINDEX with Full  Text | 230 |
| S10 | S7 OR S8 OR S9 | Expanders - Apply equivalent subjects Search modes Boolean/Phrase | Interface - EBSCOhost  Research Databases  Search Screen - Advanced  Search  Database - SocINDEX with Full  Text | 626,306 |
| S9 | "mental health" OR  Welfare OR Wellbeing  OR Well-being OR  Psychia* OR Psycho* | Expanders - Apply equivalent subjects Search modes Boolean/Phrase | Interface - EBSCOhost  Research Databases  Search Screen - Advanced  Search  Database - SocINDEX with Full  Text | 626,306 |
| S8 | DE "MENTAL health" | Expanders - Apply equivalent subjects Search modes Boolean/Phrase | Interface - EBSCOhost  Research Databases  Search Screen - Advanced  Search  Database - SocINDEX with Full  Text | 27,931 |
| S7 | DE "PSYCHOLOGICAL  well-being" | Expanders - Apply equivalent subjects Search modes Boolean/Phrase | Interface - EBSCOhost  Research Databases  Search Screen - Advanced  Search  Database - SocINDEX with Full  Text | 1,524 |
| S6 | S4 OR S5 | Expanders - Apply equivalent subjects Search modes Boolean/Phrase | Interface - EBSCOhost  Research Databases  Search Screen - Advanced  Search | 21,267 |

|  |  |  | Database - SocINDEX with Full  Text |  |
| --- | --- | --- | --- | --- |
| S5 | DE "IMMIGRATION  detention centers" | Expanders - Apply equivalent subjects Search modes Boolean/Phrase | Interface - EBSCOhost  Research Databases  Search Screen - Advanced  Search  Database - SocINDEX with Full  Text | 59 |
| S4 | removal OR detention | Expanders - Apply equivalent subjects Search modes Boolean/Phrase | Interface - EBSCOhost  Research Databases  Search Screen - Advanced  Search  Database - SocINDEX with Full  Text | 21,267 |
| S3 | S1 OR S2 | Expanders - Apply equivalent subjects Search modes Boolean/Phrase | Interface - EBSCOhost  Research Databases  Search Screen - Advanced  Search  Database - SocINDEX with Full  Text | 84,370 |
| S2 | immigration OR immigrant* or refugee* OR "asylum seeker*" OR  "foreign national offender*" | Expanders - Apply equivalent subjects Search modes Boolean/Phrase | Interface - EBSCOhost  Research Databases  Search Screen - Advanced  Search  Database - SocINDEX with Full  Text | 84,370 |
| S1 | DE "EMIGRATION & immigration" | Expanders - Apply equivalent subjects Search modes Boolean/Phrase | Interface - EBSCOhost  Research Databases  Search Screen - Advanced  Search  Database - SocINDEX with Full  Text | 23,967 |
