# Supplementary material for: How conditions within immigration removal centres in the United Kingdom affect the mental wellbeing of detainees: a scoping review
Source: J Public Health (Oxf). 2026 Jun 26;48(3):767–79. doi: 10.1093/pubmed/fdag044 (PMC13531703; doi:10.1093/pubmed/fdag044)
Supplement: Supplementary file 1 [file supplementary_material_fdag044.zip › Supplementary File E revision 2 Untracked_fdag044.docx]

Supplementary File E: Results of Included Studies Table

| Results of Included Studies (n = 19) | | | | | | | |
| --- | --- | --- | --- | --- | --- | --- | --- |
| **Author(s) (Year); Number of and Name of IRC(s); Type of Study** | | **Objective(s)** | **Population size and characteristics; methods** | **Strengths and Limitations** | **Basic Needs** | **Psychological Needs** | **Self-actualization Needs** |
| Research | Arshad, Haith-Cooper & Palloti (2018); N/S  Qual | Explore the experiences of pregnant migrant women living in detention in order to understand maternity care provision and the effect of detention on women's health. | 4 former female detainees (2 form central Europe, 1 from sub-Saharan Africa and 1 from India), experienced all or part of their first pregnancy in detention, length of detention between 53 - 212 days, and 2 midwives who volunteered in the IRC; In-depth interviews | **Strengths:**  Frequent use of textual quotes from participants. Use of two researchers in the analysis.  **Limitations:** Small sample size. Lack of interpreter use, however, notes good spoken English in participants. Policy has now changed to avoid detention of pregnant women therefore the relevance is questionable. Lack of clear presentation of all codes relating to themes. | **Basic:** Poor quality food, not enough food and strict mealtimes and unable to take food to their rooms. Felt hungry between meals.  **Safety:** Lack of staff to facilitate medical appointments, Referrals not made for mental health support. Lack of privacy from staff of any gender, including when using the toilet / shower. | **Esteem:** Not believed to be unwell, physically or mentally, by staff. Lack of privacy for medical appointments. |  |
|  | Bosworth (2014); 6 – Campsfield House, Colnbrook, Yarl’s Wood, Tinsley House, Brook House, Morton Hall  MM | 1. Use staff and detainee testimonies to revisit key assumptions about state power and the legacies of colonialism under conditions of globalisation.  2. Explore how IRCs designate certain people as strangers no matter how familiar.  3. Design a survey to capture the quality of life in detention. | N/S; Mixed methodology including ethnography and exploratory approaches. Surveys, unstructured detainee interviews, structured and unstructured staff interviews and 20 months (or over 2,400 hours) of observation and field notes | **Strengths:**  Large amount of rich ethnographic data analysed with thorough manual analysis technique. Use of translators broadened the numbers of detainees they were able to interview.  **Limitations:**  Multiple researchers meant overlap of participants and difficulty quantifying numbers and characteristics of the research population.  Potential conflict of interest through working with the Home Office as data from the research was used by them and private contractors.  Disparity between the predominant ethnographic and exploratory methodology and the generation of a survey, mixed methods although mainly quantitative in nature. Although discussion of ethical considerations during the study was noted, there is no clear evidence of approval by an appropriate body. However, this would be presumed due to its association with the University of Oxford and the Home Office. | **Basic:**  Sleep disruptions due to shared rooms and loneliness.  Some rooms with no fresh air or natural light. Varied conditions in each IRC.  Room inspections discarded personal items.  Poor-quality food, lack of cultural variety and fear of issues with meat e.g. in vegetarian food or non-Halal meat.  **Safety:**  Self-harm, food refusal and ‘dirty’ protests.  Emotional outbursts.  Fear of other detainees e.g. persecution from the cultural beliefs they are fleeing their country of origin for, racial discrimination, perceptions of FNOs, living habit issues and reports of violence within the IRCs.  Insecurity as ‘arrested’ at home or work or while registering as an asylum seeker.  Healthcare screening and help accessing lawyers given.  Difficulty accessing healthcare, potential lack of use of interpreting services causing confusion or feeling disbelieved.  Insufficient professional identity checks of lawyers permitted to visit; some detainees conned.  Hostility between detainees, custody officers and immigration staff.  Use of separation units on arrival, some without curtains, privacy and broken TVs unable to be turned off.  Even those willing to be removed, stuck in detention due to administrative errors. | **Belongingness and Love:**  Distress and anxiety from separation from children e.g. one man detained the night his wife gave birth.  Difficulty finding relatives to look after their children, some went into social care and were difficult to visit causing fear of loss of access to them permanently. Some children flown to parent’s country of origin, intending to be reunited on removal.  Children in community suffered e.g. poor behaviour or educational attainment.  Internet, phone calls, activities, faith used to stay connected or cope e.g. crafts to make gifts.  Friendships and support through other detainees e.g. Buddy system used to assist new arrivals, but emotionally challenging for the buddies.  Difficulties with other detainees – language barriers, fear of betrayals of trust, varied length of detention, cultural differences, fear of roommates.  Barriers to outside interactions – financial, geographical, immigration status, work schedules.  Confusion, mistrust and suspicion of staff and their roles. Some staff still felt they were prison officers.  Positive relationships between staff and detainees.  **Esteem:**  Loss of independence and ability to ‘live normal life’ e.g. work, find relationships.  Embarrassment of detention.  Loss of identity or in mourning of their previous life e.g. after being resident in the UK for many years.  Feelings of stigmatisation due to being an FNO.  Feeling forgotten or invisible.  Feeling dehumanised ‘like cows, like sheep’.  Feeling like prisoners who have committed no crime, slaves or victims of kidnapping causing anger. Bars on windows, shared rooms, limited time use of different standards of housing units based on merit.  Positive culture examples, detainees called ‘residents’, staff felt like care workers.  Staff generalisations of FNOs and certain nationalities.  Feeling persecuted or marginalised due to ethnicity.  Examples of dressing formally to maintain self-respect.  Challenged identity as parents  Institutionalisation, some fearing release or removal.  Loss of possessions causing embarrassment or without dignity. | Feeling lost in time.  Frustration of not knowing how long they will be detained for.  Inability to enjoy activities on offer, due to poor concentration, feeling distracted or anxious. Lack of activities on weekends, making time pass slower.  Inability to predict or plan for their futures.  Monotony of daily routine, feelings of boredom or that activities were pointless. |
|  | Gerlach (2022); 1 - Yarl’s Wood and the community  Qual | Define dignity in immigration detention and beyond.  Examine the role of dignity in detention and the way in which the existence of dignity in detention impacts on people when they are in detention and after they leave. | 76 current or previous female detainees (38 current, 12 former detainees released into the UK community, 26 former detainees removed to Jamaica); interviews | **Strengths:**  Detainees explored their own definitions of dignity. Focus groups used to aid interpretation of results into coding tables.  **Limitations:**  Author mentions reflexivity through collaboration but neglects to explore their own background and biases which may contribute to their results.  Only women included in the study which is not specified why in the objectives or methodology.  No statement on whether an ethical approval process was followed although does consider some ethical implications of the research. | **Safety:**  Difficulty accessing case workers (changing every few months) and lawyers.  Feeling neglected and/or dismissed by medical teams. Fear of not being able to access emergency medical treatments. | **Esteem:**  Lack of clarity and difficulty in navigating immigration processes led to feelings of hopelessness and helplessness with loss of autonomy. Challenges to own identity. Feelings of criminalisation - prison-like environment with barbed wire, high walls and gates, locked in the facility, and handcuffed on the plane for removal.  Feelings of loss of dignity, self-esteem and confidence which persisted even once released into the community.  Loss of confidence and work due to worsening mental health in detention persisting after release (anxiety, depression, PTSD). Living in fear of re-detention with ongoing Home Office reporting and involvement.  Embarrassment of detention. Dehumanisation - feeling like they would receive worse healthcare treatment than animals would in the UK or feeling like chickens in a slaughterhouse. | Unknown length of detention. Some preferred prison to immigration detention.  Inability to plan for the future. |
|  | Godshaw (2020); 2 - Brook House and Tinsley House  Qual | Identify routes to, and experiences of, detention for men who grew up in the UK and subsequently detained as "foreign criminals" | Phase One (Ethnographic): 24 male detainees and 20 practitioners (civil servants, NGO workers, solicitors, social worker and parliamentarian); semi-structured interviews, IRC visits, observations, document analysis  Phase Two: Case studies from 8 detainees (1 UK born, 7 born outside the UK) | **Strengths:** Multiple methods of data collection allows for triangulation. Good consenting process and ethical framework. Ethnography allows for rich insight.  **Limitations:** Personal opinions due to being a campaigner for the end to detention may have influenced the participant selection and/or findings. | **Basic:** Stress of detention caused insomnia, lack pf appetite and heart palpitations.  **Safety:** Constant uncertainty and threat of deportation to places they did not know physically or have any connection to. | **Belongingness and Love:** Challenges national and cultural belonging to the UK, disregarding socialisation and contributions to the UK for those that grew up there. Younger staff members friendlier as less of an ideological commitment to detention. Social separation in detention between those that grew up in the UK and new arrivals. Many had family bonds in the UK and detention strained their relationships.  **Esteem:** Feeling of racial discrimination from being detained itself and from staff. Seen to challenge authority when acting as "good citizens", identifying unjust treatment, helping others with immigration cases and advocating for human rights regarding conditions. |  |
|  | Griffiths (2013); 1 – Campsfield House and the community  Qual | Examine lived experiences of immigration detention and explore the relationships between detained and the British state. In particular, examine the repercussions of chronic instability on individuals and coping strategies employed. | 160 male detainees (FNOs and asylum seekers) from over 50 countries (including but not limited to Nigeria, Afghanistan, Iran, Iraq), volunteer visitors, ex-detainees, independent monitors, NGO employees, representatives from the UK Border Agency and the private company running the centre; Ethnography and interviews with the researcher working as an immigration caseworker and for a local NGO  Conducted between 2006 - 2008 | **Strengths:**  Thorough ethnographic approach taken with multiple roles within the IRC acquired and acknowledgement of the impact this may have on detainees. Mitigation technique used through informal individual-led conversations with detainees, supported by more formal interviews with various other staff members.  All analysis points reinforced with direct quotes from detainees.  **Limitations:**  Explanation of ethical guidelines followed given however not explicitly stated whether approval was sought or given by an appropriate body.  Although, a slight awareness of the researcher’s role impact on detainees and their professional background stated, minimal to no information given on the researcher’s cultural background and subsequent possible influence on the data. | **Basic:**  Over 150 detainees went on hunger strike.  Long journeys incurring sleep deprivation.  **Safety:**  Release or removals occurring at short notice, some unable to find accommodation.  Inter-detention transfers resulted in lost possessions, allegations of physical abuse, and disrupted stability. Some occurring at night and/or unexpected and unexplained resulting in fear.  Removals with unclear certainty, occurring at night, with minimal notice (<72 hours) hindering access to legal support, or multiple failed removals with unclear explanations.  Multiple fires, riots and escapes attempts causing chaos, fear and panic, especially in vulnerable detainees.  Temporal uncertainty a barrier to accessing advice/support due to not knowing where they would be day to day.  Immigration caseworkers based off site and difficult to contact, officers on site had no decision-making powers. | **Belongingness and Love:**  Inter-detention transfers disrupting support networks.  Feelings of shame, impotence and poor parenting or use of avoidance to avoid upset due to separation from family.  Social skills and relationship breakdowns after release.  Coping mechanisms through music, faith, or exercise. Usually done on an individual level, avoiding the emotional distress of other detainees.  Friendships between detainees difficult and rare due to degree of mobility, language/culture disparities.  **Esteem:**  Feelings of persecution due to nationality e.g. after one Jamaican detainee started a fire/riots, all Caribbean detainees were transferred elsewhere.  Poor post-detention removal treatment e.g. handcuffed on planes, restricted use of toilets.  Ambiguity as to reasons for detention.  Feelings of passivity and emasculation with use of strikes and privileges. One detainee thought they were given a sleeping pill to keep them docile.  Tiredness and hopelessness developed after time, resulting in passivity, feeling like ‘zombies’ or as if they were ‘dead’.  Sense of unfairness with a sudden ‘rupture’ from society. | Uncertainty around removal from detention with poor/no communication and no defined timeline or time limit for detention made it challenging for detainees to be able to plan for or imagine a future. This included both short term, such as the following days and longer term. Many preferred prisons and the criminal justice system for this reason. Fear of both sudden removal and being forgotten in detention indefinitely. |
|  | Griffiths (2014); 1 - Campsfield House and the community  Qual | How is time understood, discussed and negotiated in practice? | 160 male detainees from 50 different countries and various migration trajectories, and 160 asylum seekers in the community (aged 16 - 30) from 30 different countries - mainly Iraq, Afghanistan and Iran); interviews and ethnography with the researcher working as an immigration caseworker and volunteering with an NGO  Conducted between 2008 - 2010 | **Strengths:** Reflexivity evident throughout the report. Use of a control group of asylum seekers in the community helped to contextualise and compare findings.  **Limitations:** Interview formats/questions were not specified and presentation of findings could have been more clearly delineated in graphic/tabular format for interviews/observations etc. | **Basic:** Transfers at night disrupts sleep.  **Safety:** Frequent transfers between IRCs leads to lost possessions. Disturbances or fights occur. | **Belongingness and Love:** Transfers between IRCs disrupts ties to NGOs and legal representatives. One detainee used the time and space to form social contacts, improve fitness and stop smoking.  **Esteem:** Feeling forgotten through being left to wait. Suspended time as no timeline for release/removal. Frenzied time with quick transfers or release prohibiting time to find accommodation and becoming homeless. Transfers disrupt a detainee's plans for their day and frequent removal attempts (leaving detention and onto planes and then back into detention again). Unable to plan more than a few days ahead. Lack of control over own time. | Life has stopped, every day the same. Inability to plan means detainees struggle to imagine a future or invest in themselves - may not want to join activities / contact NGOs. |
|  | Hasselberg (2016); N/S  Qual | 1. Examine the deportation and deportability of foreign nationals convicted of one or more criminal offences in the UK.  2. Explore the way foreign nationals’ deportability is felt, understood and experienced and the strategies they deploy to cope with and react to their own deportation, or that of a close relative. | 11 former detainees (10 male, 1 female), 7 family members – cases included 7 African appellants, 3 European, 2 Latin American, 2 Caribbean, 2 South-East Asian, 1 North American, 1 Middle Eastern – all cases were FNOs; Semi-structured interviews (with former detainees/family members/legal case-workers/NGO staff/other removable migrants such as asylum seekers and visa overstayers), focus group, observations of deportation appeals at the Asylum and Immigration Tribunal. | **Strengths:**  Though not intentionally used for research purposes, the researcher’s role as a volunteer within IRCs likely helped shape interviews and develop understanding.  Thorough analysis of the researcher’s role within the study and triangulation of multiple sources of data.  **Limitations:**  No research participants were detained or imprisoned at the time of the research which may lead to recall bias. Only those who were appealing their cases were included, and only those convicted of minor offences. | **Basic:**  Shock of detention made some lose sleep, their appetite and subsequently weight.  **Safety:**  Sudden detention when reporting to the Home Office, unable to see family, children prior or collect possessions such as medicines or own clothes.  Hearing planes go by, like a constant unsettling threat of removal.  Geographical distance from their lawyers hampering legal visits.  Constant pressurisation to be removed to their country of origin, with daily questions or financial offerings.  Those who wanted and requested to be removed struggled to make it happen.  Distress caused by witnessing others who had been detained for many months or years or even others self-harming or dying by suicide. | **Belongingness and Love:**  Feelings of guilt for putting family through the difficult experience.  Difficulty seeing family due to geographical distance and subsequent expense. Feeling a lack of consideration of personal circumstances e.g. one man had a child in hospital.  **Esteem:**  Feeling a lack of trust from the government if they were to stay in the community.  Feelings of permanent criminality and feeling publicly shamed by staff for detainees and visiting family. Some spent longer in detention than in prison for their crimes. Frequent comparisons to prisons but valued being able to move freely around the detention centre during the day and have mobile phones.  Felt deprived of time and freedom for no reason. Feelings of unfairness. Feeing forgotten and dehumanised. | No release date causing depression. |
|  | Hollis (2019); Number N/S, includes Dungavel  Qual | 1. Explore and analyse the phenomenology of entering, living in and coping with life inside UK IRCs.  2. Identify psychosocial stressors in detention, psychological impact of these stressors on those detained and the way in which detainees coped with their experiences and expressed resilience. | 9 former detainees - 7 asylum seekers, 2 FNOs - 2 female, 7 male - 6 countries of origin (5 Iran, 1 Iranian-Kurdish, 1 Iraqi-Kurdistan, 1 Pakistan, 1 Yemen, Zimbabwe) - average age 31 - 24hrs to 7 months; Non-directive interviews driven by participant's concerns | **Strengths:** Use of interpreters allowed a more diverse study population. Clear phenomenological perspective. Rich depth of data supported by quotations.  **Limitations:** Self-selection bias possible. Interpreters were "supportive of the aims of the project" which may have influenced language used in the translations. Small sample size. | **Basic:** Physical and mental health needs neglected by staff (custodial and medical). Rigid routine within the IRC meant no food allowed in bedroom for a pregnant woman who struggled to eat as normal and lost weight. "What you're in" makes detainees depressed.  **Safety:** Surrounded by desperate, depressed or suicidal people. | **Belongingness and Love:** Lack of empathy from staff "made me, like very low".  **Esteem:** Detrimental to self-esteem, identity, and belief in themselves and the world. Disempowerment as unable to access translations or interpretation services when receiving immigration letters. Apparent institutional indifference to health needs. Feelings of hopelessness. Fall into rumination. Coping and resilience formed from faith, philosophy, relationships and being proactive. | Loss of meaning in life. Nothing to do in detention. |
|  | Këllezi et al (2021); 2 N/S  Qual | 1. Impact of relationships between detainees and staff on desire to seek and experiences of help.   2. Impact of detainee's perceptions of stigmatisation by staff on engagement with and experiences of health care | 40 detainees (15 men and 25 women) from "a variety of experiences, migration trajectories and time in detention" 21 staff members (9 men and 12 women) including custody officers, healthcare professionals, welfare workers, education and activity team members ; Semi-structured interviews | **Strengths:** Larger qualitative study from wide variety of backgrounds. Repetitive reflexivity throughout the study through the researcher journaling. Able to gather multiple perspectives from different viewpoints (staff vs detainee). Initial separate analysis of detainee and staff interviews helped to minimise researcher bias in the analysis. Thorough analysis phase.  **Limitations:** Deductive approach through use of the social identity framework may have influenced coding and missed new themes. | **Safety:** Fear of expressing vulnerability. | **Belongingness and Love:** Socio-political inequality and injustice felt. Staff know that detention causes distress but don't help. Staff feel helpless as the indeterminate nature of detention makes planning care difficult and don't feel that they make any difference. Bilateral mistrust between detainees and staff causing reluctance to seek help due to perceived repercussions, reactions to emotions or inadequacy of services, and limited awareness of services.  **Esteem:** Powerlessness and illegitimacy is the source of their health-related problems and distress. Detainees feel stigmatised. Help-seeking perceived as "defeat" and confirm unequal status relations. |  |
|  | Turnbull (2016); 4 - Campsfield House, Yarl's Wood, Colnbrook, Dover  Qual | Explore the theme of waiting by drawing on the lived experiences of those confined in immigration detention in the UK. | Part of larger ethnographic study exploring identity, home, and belonging:  Male and female detainees, number N/S; 149 days of observations, semi-structured interviews in-detention (n = 89) and post-detention (n = 8), focus groups (n = 3) | **Strengths:** Triangulation through ethnography and interviews/focus groups. Not clarified total numbers of participants in study and whether in-/post-detention interviews were with separate individuals or followed up.  **Limitations:** Ethnographic time mainly spent in communal areas therefore those spoken to may be coping better as able to socialise. | **Basic:** Detention provides the basic needs of food, shelter whereas for some in the community there was freedom but difficult socioeconomic conditions due to being unable to work and earn money.  **Safety:** Referred to as "kidnapping". | **Belongingness and Love:** Coping through faith or patience.  **Esteem:** Not knowing how long one will be detained for is difficult. Uncertainty of detention made detainees feel uneasy. Schedules within IRCs frustrates detainees due to lack of agency over their own time. Being stuck feels like mental torture. Feels like the government is "taking away their time", viewed as punitive and unfair. | Feel lie their lives are being "wasted", seeing others their age move on with their lives. |
|  | Zimmerman, Chatty and Nørredam (2012); N/S  MM | 1. Explore the experiences of mental and physical health needs before and during detention  2. Explore access to healthcare services while in detention | 30 former detainees - 4 female, 26 male - mean age 33 - 27 asylum seekers and 3 FNOs - countries of origin: Afghanistan (11), Iran (6), Nigeria (5), Somalia (n = 3), Morocco (n = 1), Zimbabwe (n = 1), Palestine (n = 1), Benin (n = 1), Ghana (n = 1), 10 - 990 days; structured questionnaires (adapted from the General Health Questionnaire and SF-36) and semi-structured interviews | **Strengths:** Translators used for both questionnaire and interviews increasing sample population.  **Limitations:** Recall bias may have been present as detainees were recalling mental health both before and during detention. No use of qualitative analysis software. Unclear how researchers formed agreement on analyses. | **Basic:** Health problems went untreated; was told they would call but never did. Medication taken away and changed, or unable to access whilst in detention and family unable to bring in (including HIV medication for 10 days). Some found initial health examinations effective. Felt like some doctors in detention weren't listening.  **Safety:** Re-traumatisation from detention, "making" them think too much about past experiences. | **Esteem:** Loss of confidence, feelings of worthlessness. |  |
|  | Sen et al (2018); 1 N/S  Quan | 1. Test the feasibility of screening for psychiatric morbidity in an IRC  2. Compare the mental health of a group seeking asylum to those not seeking asylum | 101 detainees in two recruitment phases, mean age 31.65, all born outside of the European Union (EU) from 27 different countries (mainly Indian subcontinent and Africa) and working knowledge of English; assessment interview employing screening questionnaires for psychiatric diagnoses and self-reporting scales for ADHD and needs assessment | **Strengths:** Attempt to mitigate selection bias through randomisation of detainees however switched to opportunistic after poor response rates. Use of validated screening tools. Inter-rater reliability assessed for all measures for researchers.  **Limitations:** Only those with proficiency in English language included in study. Limited analysis with descriptive statistics with no comparisons between sub-groups such as country of origin / age etc. | **Basic:** Needs met for accommodation (70.4%), food (77.2%), looking after living environment (79.2%). Unmet needs included psychological distress (72.3), daytime activities (45.5%).  **Safety:** Safety to self-unmet need for 20.8%. | **Belongingness and Love:** Unmet need of company (62.4%), intimate relationships (76.2%), sexual expression (71.3%). Met need of telephone (96%). |  |
|  | Sen et al (2021); 1 N/S  Quan | Identify whether foreign national prisoners in one IRC differed from other detainees in screened prevalence rates of mental disorder, levels of unmet needs and length of stay. | Secondary data analysis from previous study Sen et al (2018):  101 detainees in two recruitment phases, mean age 31.65, all born outside of the European Union (EU) from 27 different countries (mainly Indian subcontinent and Africa) and working knowledge of English; assessment interview employing screening questionnaires for psychiatric diagnoses and self-reporting scales for ADHD and needs assessment | Same data set as Sen et al (2018) therefore similar strengths/limitations  **Limitations:** No p values for comparisons between FNOs and "other detainees", therefore statistical significance unknown. | **Basic:** Similar proportions of met needs for living (food, accommodation, health).  **Safety:** Higher unmet needs for drugs and alcohol (21.4% and 10.7%) for FNOs compared to other detainees where there was mainly no need or need not applicable (95.5% and 95.5%). | **Belongingness and Love:** Higher unmet needs for FNOs (89.3%) in intimate relationships than other detainees (71.2%). |  |
|  | Lungu-Byrne et al (2021); N/S  SR | What is known about migrant health experience and unique support needs in European prisons and immigration detention settings since commencement of the European migrant crisis? | 3 UK research studies comprised a total of 114 detainees (or former), 2 healthcare professionals; scoping review of European studies (involving interviews and questionnaires), editorials, articles, correspondence | **Strengths:** Good range of databases searched. Use of inductive coding allows further insight into data.  **Limitations:** Search terms in search strategy unclear / limited with no database thesaurus terms used. Thematic analysis is better suited to systematic reviews rather than scoping. | **Basic:** Unable to take food to rooms even if expressing hunger to staff.  **Safety:** Lack of mental health referrals due to lack of trained staff, language barriers, and lack of culturally competent care. Lack of continuity of care post-release. | **Belongingness and Love:** Poor communication and lack of adequate translations when receiving legal letters. |  |
|  | Van Hout, Lungu-Byrne and Germain (2020)  SR | What is known in the qualitative literature about migrant detainee's health experience and unique healthcare needs when detained in European immigration detention settings? | Total of 37 detainees (6 pregnant) and 15 staff; Scoping study of 2 UK studies and 2 Swedish studies all using interviews | **Strengths:** Comprehensive search strategy.  **Limitations:** Small sample size for a scoping review. Thematic analysis better suited to systematic review as more reliable due to rigorous methods. | **Basic:** Poor quality food and strict mealtimes and no choice of food, particularly difficult for pregnant women.  **Safety:** Lack of privacy, feeling unsafe with abusive and controlling staff behaviour. Awareness of peer suicide attempts. Poor continuity of care within the IRC from cancellations of external appointments from staff shortages and shortage of midwives in the centre. Disrupted provision of medication. Lack of privacy when receiving healthcare. | **Belongingness and Love:** Poor communication / translation facilitation, some detainees unable to understand where they were or why. Some felt shame so didn't tell their families when they first arrived. Staff unaware of the impact of detention on detainee's mental health. Not feeling listened to when expressing concern over one's health.  **Esteem:** Loss of identity. Hopelessness. Sense of powerlessness over one's health. |  |
| Grey | Bosworth and Gerlach (2020); 7 - Brook House, Tinsley House, Harmondsworth, Colnsbrook, Morton Hall, Yarl's Wood, Dungavel  CS | Measure detainee's perceptions of the IRC in which they are residing including their views on their immigration case, mental health and their overall quality of life. | 459 current male and female detainees of 88 different nationalities - mean age of 35 (range 19 to 82) - mainly of Christian or Muslim faith - 55% had been in detention longer than 28 days - 82.3% lived in the UK longer than 1 year prior to detention; mixed methods survey | **Strengths:** Large scale project across all IRCs at the time and attempting to survey all detainees. Use of a context-specific instrument with longitudinal use in other IRCs facilitating comparison between centres. Ethical safeguards such as anonymisation, accessibility through translations and voluntary participation.  **Limitations:** Self-selection bias is possible, Response rate of 33% in total. Lack of a statement locating the researchers culturally and institutionally although implied by the affiliation with the University of Oxford and Centre for Criminology. Only given 2 days to answer the survey. Limited number of survey translations and requests for Kurdish, Punjabi, Portuguese, Romanian and Farsi unable to be facilitated. Not indicated if any instances where unable to approach detainees and therefore real response rate unclear. Results not delineated into gender. Use of means for ordinal data inappropriate as implies measures between categories are equal. | **Basic:** Food good sometimes (39%) or never (40%). Only 46% said they had enough clothes most / all of the time. 55% said centre was clean sometimes or never. 60% said enough activities sometimes or never.  **Safety:** 62% of detainees had thought about ending their life or hurting themselves all or most of the time in the 7 days prior to the survey yet only 10% said they had been on an ACDT. Perceptions of poorer healthcare standards than externally and difficulty accessing it. | **Belongingness and Love:** Many listed talking to friends/other detainees, gym/sports and visiting the library as activities undertaken and many said this made them feel good. However, 18% said they spent most of their time doing "nothing" and 38% believed activities they did, did not make them feel good. Of the 60% of detainees with friends/family in the UK, 52% reported it to be hard for them to visit. 19% of detainees did not have any family/friends in the UK that could come visit them. 28% spoke to "nobody" when they were upset. Of the 50% that require interpretation services, 35% were unable to access it. 22% believed staff made racist comments. Better scores for relationships between detainees, however a third reported detainees use illegal drugs which cause problems and 60% spent most of their time alone.  **Esteem:** Of all those who had raised a previous complaint within their centre, 81% were not satisfied with how it was handled. Only 50% believed they were being treated as humans in the IRC. 69% said officers treated them with respect always / most of the time compared to 52% for immigration officers. |  |
|  | Canning (2019); N/S  Qual | Empirically identify the harms embedded in asylum systems in Britain, Denmark and Sweden using typologies of harm. | N/S number of women in asylum accommodation in North West England and N/S number of "practitioners" in London; semi-structured interviews | **Strengths:** Clear philosophical perspective and rationale behind methodology and methods.  **Limitations:** Structure of results confusing and methods could be more detailed relating to participant inclusion. | **Basic:** Poor medical facilities. Problems with women accessing appropriate sanitation or able to choose between tampons and sanitary towels.  **Safety:** Barriers to accessing lawyers within the minimum 3-day time frame of removal, particularly when moving between Scotland and England. Lack of privacy/dignity, guards bursting into rooms in inappropriate situations for the detainee. | **Esteem:** Temporal uncertainty distressing. Psychological effects of discrimination as only there because one is an immigrant. Gender differences for those victim of sexual abuse e.g. female detainees with male detention officers in charge, re-traumatising. |  |
|  | Centre for Mental Health (2017); 8 - The Verne, Morton Hall, Yarl’s Wood, Campsfield House, Brook House, Tinsley House, Harmondsworth, Colnbrook  MM | 1. Provide a rapid mental health needs analysis  2. Describe mental health need, existing provision and gaps in service  3. Comment on relevant aspects of IRC regimes  4. Make recommendations for improvement and address gaps | Review of the literature, secondary data analysis, cross-sectional survey of 35 detainees completed by mental health staff at 4 IRCs and cross-sectional survey of health care managers, interviews with 32 detainees and staff and key stakeholders - including managers, practitioners and NGO members (number N/S), observations | **Strengths:** Mixed methodology with a large variation in sources of data allows for triangulation.  **Limitations:** Small sample size for the population sampled (30/2,654). Numbers of staff / key stakeholders interviewed and surveyed are not specified. Sample characteristics for the Health of the Nation Outcome Scale survey shows a total of 55 participants however stated within the text as 35. No standardisation of results, as some figures markedly higher/lower than others. | **Basic:** 24-hour reception and therefore admission throughout the night causing sleep deprivation and distress, especially for those more vulnerable e.g. older adults. Occurs frequently due to wanting to avoid daytime traffic.  **Safety:** Locks and no fresh air made it feel suffocating, like captivity which is re-traumatising for some. Some commented positively on accessible healthcare. Lack of trained staff to deliver regular psychological interventions. Difficult and untimely transfer of vulnerability information from their previous place of living e.g. community/prison/hospital and frequent transfers. "Application" processes to seek healthcare felt to delay care. Poor training for staff within ACDT team. Delays in transfer to hospital for mental health due to shortages of hospital beds, and use of separation unit whilst in IRC increased paranoia and isolation. Removal decisions often communicated at the last minute as staff felt it would increase risk of self-harm/suicide if given prior notice, meaning that mental health staff had little or no time to implement coping strategies or link with support systems. Rapid release makes it difficult/impossible for mental health staff to ensure continuity of care, especially over a weekend. Inadequate facilities / equipment for mental health services. High rates of DNAs for mental health appointments due to transiency of detainees, legal meetings taking priority, staff shortages limiting transfer ability for external appointments, limited in-house facilities and patient engagement. Some detainees scared of officers due to manhandling meaning less likely to seek help. Better to "keep your head down". Substance misuse a problem in some parts of IRCs. LGBT detainees fear of revealing their sexuality due to other detainees expressing homophobic views, especially within own ethnic/cultural group. Aggressive outbursts met with sanction rather than addressing cause of distress. Staff shortages prevent being able to carry out healthcare recommendations such as constant observation. | **Belongingness and Love:** Social interactions difficult due to ever-changing and distressed population leading to isolation. Mental health nurses help build coping strategies for distress. Staff encouragement to participate in activities helped manage stress. Well-being sessions including talking therapies, massages, meditation, were well-received and helped with relaxation. Where it was possible, peer support found to be helpful. Chaplaincy very helpful for cultural/religious support. Detainees don't feel listened to by staff, but understand it' because the staff are trying to help but the system is preventing them. FNOs (transferred from prison) may seem threatening to other detainees due to prison mentality / culture. Staff becoming desensitised to detainees’ distress as a coping mechanism for their own mental well-being.   **Esteem:** Loss of liberty due to prison-like regime (locked up at night, no choice over roommate, restricted activities and time schedules). Uncertainty about their future, undetermined time in detention distressing - Lack of ability for good achievements to be recognised and in turn impact their detention (e.g. early release in prison). Mental health support discouraged detainees from mentioning their legal cases in sessions, restricting their ability to vent. Lack of staff (including mental health) training/supervision therefore continued disbelief in detainee accounts, poorer quality of care and lack of debriefing after untoward incidents. | Lack of purpose - in prison there were jobs and a prison council. |
|  | Gallagher (2017); 3-4, Yarl's Wood, Harmondsworth, 2 N/S  Qual | Investigate how immigration detention impacts mental health, with consideration for psychosocial processes that contribute to harm or resilience | Qualitative study for a Thesis  10 former detainees (7 women and 3 men) - ages 30 - 55 - 8 asylum seekers and 2 long-term residencies (4 FNOs) - length of detention ranged from less than 1 week to over 2.5 years; semi-structured interviews using grounded theory | **Strengths:** Detailed methodology and with data collection and analysis and thorough ethical approach. Codes presented in a table with accompanying quotes.  **Limitations:** One female interviewer and recruitment through only one source (charity offering therapeutic interventions) therefore may be participant bias. Not documented when interviews took place for former detainees, therefore unable to ascertain risk of recall bias. Detention occurred between 2009 and 2014 therefore conditions may have changed since then. | **Basic:** No control over environment - who they shared rooms with, noise levels, warmth levels, diet. Disrupted sleep e.g. by staff or by the distress. Some become unable to eat and often vomited.   **Safety:** Invasions of privacy, staff bursting into rooms unannounced. Lack of information into their detention / how to apply for bail/accommodation/asylum. Rumours about sexual misconduct within the IRC. Feeling persecuted by particular staff or threatened by staff (of deportation) and/or other detainees with an example of sexual assault. Trauma of detention in their thoughts post-detention. | **Belongingness and Love:** Fear of monitoring by staff meant detainees felt unable to have private conversations with those outside detention. Monitored through head counts, cameras, checks. Told emails are checked. Restrictions to communicating with the outside world. Some detainees helped each other, although there were language barriers. Some cope through religion or staying active.  **Esteem:** Loss of agency/freedom due to regime and being locked up, phones taken off them and restricted access to internet. Not feeling listened to/believed/cared for e.g. when discussing concerns with staff like bedbugs or when unwell or seeing other vulnerable detainees (dehumanising). Uncertainty of detention contributed to feeling a loss of control and lives being in limbo. Feeling criminalised and persecuted when they don't know what they've done wrong. To cope, some "fought back" through challenging staff, protesting and in turn feeling empowered. Memory was affected for some forgetting short-term events. | Loss of identity due to loss of previous role, nationality, belongings. |
|  | HMIP (2022); Derwentside  MM | Inspection to assess outcomes for detainees against four healthy establishment tests: safety, respect, activities, and preparation for removal and release. | 21 female detainees, aged between 21 and 59, detention length between less than a week to 6 months; Observations, detainee and staff mixed-method surveys, discussions with detainees / staff / relevant third parties, semi-structured interviews with 3 detainees, documentation | **Strengths:**  Detainee survey available in 23 different languages and there was a 78% response rate. Survey used clear, inspection criteria according to human rights standards.  **Limitations:**  Results compared to prisons. Only 41 out of 224 staff members completed the survey and it may have been completed multiple times by the same staff member. Little reference to confounding factors and the researcher’s (inspector’s) influence on the detainee’s answers and the research. Lack of representation of qualitative data and therefore detainee’s voices and contextual viewpoints suppressed to an extent. | **Basic:**  Complaints regarding food and lack of cultural diversity. Scarcity of healthy food options and access to hot food poor. Access to recreational areas for 14 hours per day, not locked in rooms at night.  Some staff showed minimal awareness of the needs of women. Good living conditions with large, clean and well-ventilated rooms.  **Safety:**  Detainees at risk of self-harm or suicide did not receive consistent or well-organised care. 45% reported having felt unsafe in the unit.  Use of force not always professionally conducted.  Use of separation unit not always used appropriately.  Systems to address behaviour concerns inadequate.  Many arrived late at night and little use of interpreting services on arrival.  Good access to Home Office staff. Geographical constraints for legal in-person visits. Good healthcare provision including access to dentists and pharmacies in place. Healthcare staff screened for women’s health issues such as female genital mutilation and pregnancy, identifying those at risk, and used interpreting services well.  No welfare staff training although detainees still found them helpful. Leadership not proactive at identifying more isolated detainees who would benefit from visiting volunteers. 65% reported no one had helped them prepare for leaving the IRC. | **Belongingness and Love:**  Poor mobile phone reception made contacting relatives difficult.  Unfinished building work restricted activities on offer.  Examples of disrespect from staff and poor understanding of detainee’s past traumas or present concerns. 95% of detainees had not had visits from family or friends since arriving.  **Esteem:**  All detainees felt respected by staff. Although there was witnessed inappropriate language and lack of compassion from staff.  Staff entered detainees’ rooms at night with inadequate justification.  Access to facilities for worship. | Educational staff and activities provided but minimal range and insufficient materials for English teaching.  Provision of 18 paid jobs, library books mainly in English, gym and sports hall. |
|  | HMIP (2023); Tinsley House  MM | 1. Inspection to assess outcomes for detainees against four healthy establishment tests: safety, respect, activities, and preparation for removal and release.  2. Comment on leadership in the establishment. | Observations, 28 detainee surveys (9 Asian, 2 Black, 1 Mixed, 16 White) and 25 staff surveys, 9 detainee semi-structured interviews (6 different nationalities), staff and relevant third-party interviews, review of documentation | **Strengths:** Large team of inspectors and researchers. Surveys conducted in wide range of languages increasing accessibility.   **Limitations:** Staff survey able to be completed multiple times by the same device therefore may have been completed multiple times by the same staff member. Detainee survey distributed and collected on the same day and staff only given 7 days to complete it. Compared to prisons. No evidence of staff discussions or the documentation reviewed. | **Basic:** Clean living conditions. Issue with ventilation so detainee's rooms very hot and non-opening windows. Limited storage space. 96% could shower every day, reported as clean but drains smelt foul. Noise levels at night an issue, only 73% said it was quiet enough to sleep and some described other detainees causing noisy behaviour. 92% had enough clean clothes. Belongings stored at reception and 81% said they could access it when needed. 80% said the food was good. Meals restricted to the dining room and halal and non-halal food stored together. 33% self-reported a physical disability but no adapted accommodation available. Able to access recreational areas/outside spaces for 13.5 hours per day and between these times confined to their corridors. Some detainees released as homeless and lack of safeguards for release of vulnerable detainees.  **Safety:** 75% felt safe their first night in detention. Poor communication between staff of adults at risk, with one detainee not having a care plan until 4 months later when found researching how to commit suicide. Night-time observations for detainees not at risk unnecessary/intrusive. Poor communication between IRCs including about at-risk adults with lack of transfer of ACDT documentation. 17% said they felt unsafe. No longer using handcuffs routinely for transfers to hospital. Able to access cigarettes but not vapes, therefore those that vaped either had to give up or start smoking. Limited health information in languages other than English and some available resources e.g. barrier protection not advertised. Health and well-being day being planned. Nurse and GP appointment available every day. Easy access to mental health teams and drug and alcohol misuse teams, pharmacists. | **Belongingness and Love:** Diversity and inclusion events only advertised in English. No detainee representatives. Although available, useful translated information not always issued to detainees. Interpretation services underused when only 43% reported to understand spoken English and 50% written English. Only 63% said they could speak to a Chaplain of their faith in private. Unable to access social media. Bookings required to visit detainees, however transport to and from the local train station provided for free for visitors. Strict visiting restrictions regarding physical touch, only 6 detainees could have visitors at one time. Smartphones prohibited in the IRC and replaced with basic mobiles. Only one free letter could be posted per detainee per week.  **Esteem:** Centre welfare team active in helping to find legal representatives. Helpful websites pinned to homepage of computers. Broken video-link technology so unable to use for bail hearings. Most staff polite and respectful. Feedback forums held weekly but resultant information only available in English. 55% said they had enough to do. Only English language and Art were the education activities available and taught at the same time by the same teacher, poor educational monitoring. Paid work by detainees was remunerated at £1 per hour. Library available but no qualified librarian, therefore staff unable to help and shortage of appropriate books e.g. language, level of learning, genres. DVDs available but no DVD player. Shortage of working computers. Welfare team available but many unaccredited. Transfers to other IRCs done at short notice and at night and detainees not always told the reason for the transfer. Poor communication about removal / release. Staff neglect to knock before entering rooms. |  |
|  | Lawlor and Sher (2016); N/S  Qual | How is mental health managed in IRCs? | Senior management, caseworkers, healthcare staff, detention staff and detainees (numbers N/S); interviews | **Strengths:** Triangulation between different stakeholders.  **Limitations:** Poor explanation/disjointed display of methods, numbers of participants, method of participation e.g. interviews/surveys/focus group, response rates etc. | **Safety:** Shortages in detention staff (custody and healthcare) leading to mistakes with medication. | **Belongingness and Love:** Lack of emotional support from staff.   **Esteem:** Poor communication between officers and other stakeholders e.g. about scheduled interviews, detainee's statuses, reviews and final decision-making. Reports of racism from staff. Feel like prisoners, helpless and vulnerable. |  |
|  | Right to Remain (2019); N/S  NS | A rough guide to immigration detention for people at risk and their supporters | No sample size as commentary drawing on accounts from "experts-by-experience", text from Immigration Bail Project Scotland, and the Right to Remain Toolkit, a self-help and mutual support guide to the UK immigration and asylum system and procedures. | **Strengths:** Use of quotations and pictorials. States where information was derived from.  **Limitations:** The nature of being grey literature limits its reliability. No comments on individual IRCs and their systems. Not identified which IRC the writer / experts were held in. | **Basic:** Phone taken off detainees and replaced with basic handset, previous SIM may not work. Noisy environment, especially at night with keys, whistling, doors banging.  **Safety:** Delays in medication due to being replaced by prescriptions within the IRC. Being taken to an IRC in the middle of the night. Security doors between different areas of IRCs and possibility of being searched by staff. | **Belongingness and Love:** Staff may not be able to help with information about procedure of detention or detainees’ rights. Poor communication - not informed why they may be transferred between centres. If have no one to visit then detainees may feel like they belong in the IRC. Visitors help them feel human and connected to the outside world. Religious support helped detainees. Visitor restrictions - need 24hrs notice, ID and have their photo and fingerprints taken. Prayer groups organised by detainees.  **Esteem:** Travelling to IRC in a secure van "like prisoners". Deprivation of liberty with passport/bank statements confiscated ("could make fake ID"). Loss of privacy with "lots" of cameras and specified regime. Limited access to computers and therefore internet and some websites (Facebook, skype) blocked. Feeling like you have done something wrong or unwanted. "You can detain the body but not the mind". |  |
|  | UKLIG & Stonewall (2016); N/S  Qual | N/S | 22 lesbian, gay, bi and trans former detainees (asylum seekers) - all held within the previous 3 years for between 3 days and 18 months - from 11 different countries including Asia, Africa, the Caribbean and Russia; in-depth interviews | **Strengths:** Rich data analysis with significant use of quotations to support statements.   **Limitations:** No clear objective stated. Not stated how many/which IRCs detainees had been held in, possibly for anonymity purposes. | **Basic:** Instances of mental health concerns being dismissed by healthcare staff or difficulties accessing medication as it is confiscated from detainees on arrival at IRCs including HIV medication and transition treatment causing physical health issues and severe mental distress.  **Safety:**  Trans detainees face threats of violence. Staff (those responsible for their safety) are seen to fail to protect LGBT detainees from abuse, often lacking basic understanding of LGBT issues or displaying discriminatory attitudes. Post-detention - Fear for their own safety if they were to return post-detention, some believing they would commit suicide. Surrounded by trauma - "they found a woman in the bathroom dead". Lack of privacy, instances of male staff entering the rooms of female detainees unannounced whilst they were naked. | **Belongingness and Love:** Feel forced to hide gender identity and sexual orientation to avoid discrimination and harassment. Feelings of isolation, high walls and only allowed outside at certain times.  **Esteem:** Lack of privacy to discuss intimate issues regarding their LGBT case. Difficulty accessing outside resources such as communicating with families/ banks and accessing websites relevant to their case as many websites blocked e.g. about the LGBT situation in their country of origin. Leads to lack of evidence for their asylum claim. Staff lacking respect for detainees, being intimidating and exhibiting discrimination |  |
|  | Notes: | Qual = Qualitative  Quant = Quantitative  CS = Cross-sectional  MM = Mixed Methods  NS = Non-standard (i.e. unable to be classified into a standard research category)  SR = Scoping Review | | | | | |
