# Supplementary material for: How conditions within immigration removal centres in the United Kingdom affect the mental wellbeing of detainees: a scoping review
Source: J Public Health (Oxf). 2026 Jun 26;48(3):767–79. doi: 10.1093/pubmed/fdag044 (PMC13531703; doi:10.1093/pubmed/fdag044)
Supplement: Supplementary file 1 [file supplementary_material_fdag044.zip › Supplementary File F revised Untracked_fdag044.docx]

Supplementary File F: Critical Appraisal Checklists

| **JBI Critical Appraisal Checklist for Qualitative Research** | | | | | | | | | | |  |
| --- | --- | --- | --- | --- | --- | --- | --- | --- | --- | --- | --- |
|  | | | | | | | | | | |  |
| **Author(s) (Year)** | **Is there congruity between the stated philosophical perspective and the research methodology?** | **Is there congruity between the research methodology and the research question or objectives?** | **Is there congruity between the research methodology and the methods used to collect data?** | **Is there congruity between the research methodology and the representation and analysis of data?** | **Is there congruity between the research methodology and the interpretation of results?** | **Is there a statement locating the researcher culturally or theoretically?** | **Is the influence of the researcher on the research, and vice-versa, addressed?** | **Are participants, and their voices, adequately represented?** | **Is the research ethical according to current criteria or, for recent studies, and is there evidence of ethical approval by an appropriate body?** | **Do the conclusions drawn in the research report flow from the analysis, or interpretation, of the data?** | **Total Score:** |
|  |  | | | | | | | | | |  |
| Arshad, Haith-Cooper & Palloti (2018) | Y | Y | Y | Y | Y | Y | N | Y | Y | Y | 90% |
| Bosworth (2014) | Y | Y | Y | Y | Y | Y | Y | Y | N | Y | 90% |
| Canning (2019) | Y | N | Y | Y | Y | Y | N | N | N | Y | 60% |
| Centre for Mental Health (2017) | Y | Y | Y | Y | Y | N | N | Y | N | Y | 70% |
| Gallagher (2017) | Y | Y | Y | Y | Y | Y | Y | Y | Y | Y | 100% |
| Gerlach (2022) | Y | Y | Y | Y | Y | N | N | Y | N | Y | 70% |
| Godshaw (2020) | Y | Y | Y | Y | Y | Y | N | Y | Y | Y | 90% |
| Griffiths (2013) | Y | Y | Y | Y | Y | N | N | Y | N | Y | 70% |
| Griffiths (2014) | Y | Y | Y | Y | Y | N | N | Y | N | Y | 70% |
| Hasselberg (2016) | Y | Y | Y | Y | Y | Y | Y | Y | Y | Y | 100% |
| Hollis (2019) | Y | Y | Y | Y | Y | Y | N | Y | Y | Y | 90% |
| Këllezi et al (2021) | Y | Y | Y | Y | Y | Y | N | Y | Y | Y | 90% |
| HMIP (2022) | N | Y | Y | N | N | Y | N | N | N | Y | 40% |
| HMIP (2023) | N | Y | Y | N | N | Y | N | N | N | Y | 40% |
| Lawlor and Sher (2016); N/S | Y | Y | Y | Y | Y | Y | N | N | N | Y | 70% |
| Turnbull (2016) | Y | Y | Y | Y | Y | N | N | Y | N | Y | 70% |
| UKLIG & Stonewall (2016) | Y | N | Y | Y | Y | Y | N | Y | N | Y | 70% |
| Zimmerman, Chatty and Nørredam (2012) | Y | Y | Y | Y | Y | Y | N | Y | Y | Y | 90% |
| Notes: | Y = Yes N = No | | | | | | | | |  |  |
|  |  |  |  |  |  |  |  |  |  |  |  |
|  |  |  |  |  |  |  |  |  |  |  |  |

| **JBI Critical Appraisal Checklist for Prevalence Studies** | | | | | | | | | | |
| --- | --- | --- | --- | --- | --- | --- | --- | --- | --- | --- |
|  | | | | | | | | | | |
| **Author(s) (Year)** | **Was the sample frame appropriate to address the target population?** | **Were study participants sampled in an appropriate way?** | **Was the sample size adequate?** | **Were the study subjects and the setting described in detail?** | **Was the data analysis conducted with sufficient coverage of the identified sample?** | **Were valid methods used for the identification of the condition?** | **Was the condition measured in a standard, reliable way for all participants?** | **Was there appropriate statistical analysis?** | **Was the response rate adequate, and if not, was the low response rate managed appropriately?** | **Total Score:** |
|  |  | | | | | | | | |  |
| Bosworth and Gerlach (2020) | Y | Y | Y | Y | Y | Y | Y | Y | Y | 100% |
| Centre for Mental Health (2017) | Y | Y | N | N | Y | Y | Y | N | N | 56% |
| Sen et al (2018) | Y | Y | Y | Y | Y | Y | Y | N | N | 78% |
| Zimmerman, Chatty and Nørredam (2012) | Y | Y | Y | Y | Y | Y | Y | Y | Y | 100% |
| Notes: | Y = Yes N = No | | | | | | | | | |
|  |  |  |  |  |  |  |  |  |  |  |
|  |  |  |  |  |  |  |  |  |  |  |

| **JBI Critical Appraisal Checklist for Cross-sectional Studies** | | | | | | | | | |
| --- | --- | --- | --- | --- | --- | --- | --- | --- | --- |
|  |  |  |  |  |  |  |  |  |  |
| **Author(s) (Year)** | **Were the criteria for inclusion in the sample clearly defined?** | **Were the study subjects and the setting described in detail?** | **Was the exposure measured in a valid and reliable way?** | **Were objective, standard criteria used for measurement of the condition?** | **Were confounding factors identified?** | **Were strategies to deal with confounding factors stated?** | **Were the outcomes measured in a valid and reliable way?** | **Was appropriate statistical analysis used?** | **Total Score:** |
|  |  | | | | | | | |  |
| Bosworth (2014) | N | Y | Y | Y | Y | N | Y | Y | 75% |
| HMIP (2022) | Y | Y | Y | Y | N | N | Y | Y | 75% |
| HMIP (2023) | Y | Y | Y | Y | N | N | Y | Y | 75% |
| Sen et al (2021) | Y | Y | Y | Y | Y | N | Y | Y | 88% |
| Notes: | Y = Yes N = No | | | | | | | | |
|  |  |  |  |  |  |  |  |  |  |
|  |  |  |  |  |  |  |  |  |  |

| **JBI Critical Appraisal Checklist for Evidence Synthesis** | | | | | | | | | | | | |
| --- | --- | --- | --- | --- | --- | --- | --- | --- | --- | --- | --- | --- |
|  | | | | | | | | | | | | |
| **Author(s) (Year)** | **Is the review question clearly and explicitly stated?** | **Were the inclusion criteria appropriate for the review question?** | **Was the search strategy appropriate?** | **Were the sources and resources used to search for studies adequate?** | **Were the criteria for appraising studies appropriate?** | **Was critical appraisal conducted by two or more reviewers independently?** | **Were there methods to minimise errors in data extraction?** | **Were the methods used to combine studies appropriate?** | **Was the likelihood of publication bias assessed?** | **Were recommendations for policy and/or practice supported by the reported data?** | **Were the specific directives for new research appropriate?** | **Total Score** |
|  |  | | | | | | | | | | |  |
| Lungu-Byrne et al (2021) | Y | Y | Y | Y | N | N | Y | Y | N | Y | Y | 73% |
| Van Hout, Lungu-Byrne and Germain (2020) | Y | Y | Y | Y | N | N | N | Y | N | Y | Y | 64% |
| Notes: | Y = Yes N = No | | | | | | | | | | | |
|  |  |  |  |  |  |  |  |  |  |  |  |  |
|  |  |  |  |  |  |  |  |  |  |  |  |  |

| **Grey Literature Critical Appraisal OCEBM** | | |
| --- | --- | --- |
|  | | |
| **Author(s) (Year)** | **Article design and methodology** | **Level of Evidence** |
|  |  |  |
| Right to Remain (2019) | Leaflet drawing upon expert opinions | 5 – Expert Opinion |
