# Supplementary material for: How conditions within immigration removal centres in the United Kingdom affect the mental wellbeing of detainees: a scoping review
Source: J Public Health (Oxf). 2026 Jun 26;48(3):767–79. doi: 10.1093/pubmed/fdag044 (PMC13531703; doi:10.1093/pubmed/fdag044)
Supplement: Supplementary file 1 [file supplementary_material_fdag044.zip › Supplementary_File_D_Revised_fdag044.docx]

Supplementary File D: PRISMA Flowchart adapted by Dr N Gallagher from Page, McKenzie (1)

Reports not retrieved as full-text not available

(n = 15)

Records sought for retrieval of full text

(n = 107)

Online records identified from:

Governmental organisations (n = 10)

Non-governmental organisations or charities

(n = 5)

Citation Searching (n = 5)

Records identified from databases:

CINAHL Plus (n = 289)

EMBASE (n = 475)

MEDLINE (n = 367)

PsychINFO (n = 437)

SocINDEX (n = 230)

Global Health (n = 102)

Records removed *before screening*:

Duplicate records removed by EndNote and manual search

(n = 738)

Records screened by title and/or abstract

(n = 1162)

Records excluded based on eligibility criteria

(n = 1049)

Reports assessed for eligibility

(n = 92)

Reports excluded:

Lack of distinct evaluation of UK IRCs (n = 41)

Lack of detainee’s own experiences of conditions in IRC

(n = 40)

**Identification of studies via databases and registers**

**Identification**

**Screening**

Reports included in review

Studies (n = 15)

Grey literature (n = 9)

**Included**

**Identification of grey literature**

Reports sought for retrieval of full text from:

Online records (n = 20)

Databases (n = 6)

Reports assessed for eligibility

(n = 26)

Reports excluded:

Lack of distinct evaluation of UK IRCs

(n = 2)

Lack of detainee’s own experiences of conditions in IRC

(n = 6)

Not two most recent IRC inspections

(n = 5)

1.	Page MJ, McKenzie JE, Bossuyt PM, Boutron I, Hoffmann TC, Mulrow CD, et al. The PRISMA 2020 statement: an updated guideline for reporting systematic reviews. BMJ. 2021;372:n71.
